# Illuminating the complexities of conflict with evolution: validation of the scales of evolutionary conflict measure (SECM)

**DOI:** 10.1186/s12052-020-00137-5

**Published:** 2020-11-23

**Authors:** Gena C. Sbeglia, Ross H. Nehm

**Affiliations:** 1 Department of Ecology and Evolution, Stony Brook University, Stony Brook, NY 11794 USA; 2 Department of Ecology and Evolution, Program in Science Education, Stony Brook University, Stony Brook, NY 11794 USA

**Keywords:** Conflict, Acceptance, Evolution, Religiosity, Psychometrics, Instrument, Validation

## Abstract

**Background:**

Although personal, familial, and community conflict with evolution have been documented in the literature, these scales require conceptualization as a construct and operationalization as a measure. The Scales of Conflict with Evolution Measure (SECM) instrument was developed in response to these needs. Using a construct validity framework, the content, internal structure, convergent, and substantive validity of the SECM were evaluated using Rasch analysis, Structural Equation Modeling (SEM), and follow up questioning. The conceptual utility of the instrument was explored by examining whether it added explanatory insights into evolution acceptance above and beyond religiosity, evolution knowledge, and background variables.

**Results:**

A literature review and expert consultation indicated that construct of evolutionary conflict perception should (i) encompass the hierarchical nature of human social structures (personal, family, community) and (ii) probe conflict as it relates to human values, cultures, and beliefs. A three-dimensional construct was operationalized as a nine-item rating scale measure. Using Rasch analyses of SECM responses from a diverse sample of > 1000 students studying evolution, the instrument met criteria of robust measurement, including: fit to model expectations; three-dimensional structure; high reliability; good rating scale function; measurement invariance with time; and convergence with a similar construct. SEM showed that: (i) family and community conflict had unique causal contributions to personal conflict, with family showing a stronger and modest impact, and (ii) personal conflict had a significant and modest causal impact on evolution acceptance above and beyond the contributions of religiosity, evolution knowledge, and background variables.

**Conclusion:**

The SECM is an easy-to-administer instrument to measure conflict with evolution and is supported by several forms of validity evidence. The SECM has potential for facilitating measurement of evolutionary conflict in educational settings, thereby raising instructor awareness of conflict levels in students, promoting rigorous evaluations of educational interventions designed to reduce conflict, and fostering conceptual advances in the field of evolution education. Future work is needed to gather additional forms of validity evidence and to test current validity claims in additional participant samples. SECM measures should also be incorporated into more complex SEM models that treat evolution knowledge and religiosity as part of the structural paths to evolution acceptance. Such models could provide insights into the most worthwhile targets for the development of educational interventions to mitigate conflict at multiple scales.

## Introduction

The idea that individuals may harbor feelings of conflict with evolutionary principles, and that such conflict may be linked to their acceptance of evolution, has been discussed in the literature for decades (e.g., Clough 1994; Dagher and BouJaoude 1997; Graves 2019; Nehm et al. 2009; Scharmann and Harris 1992; Turner 1978). Many qualitative studies (and a few quantitative ones) confirm that feelings of conflict are an important variable to consider in the complex web of factors accounting for evolution acceptance (e.g., Barnes et al. 2020; Clough 1994; Ha et al. 2012; Konnemann et al. 2018; Nehm et al. 2009; Scharmann and Harris 1992). Conflict with evolution may be characterized in many ways: by its presence (i.e., yes, no), magnitude (high, low), sources (e.g., religion, societal implications), scales (e.g., personal, family, community), and consequences (e.g., anxiety, avoidance) (Barnes et al. 2020; Clough 1994; Dagher and BouJaoude 1997; Konnemann et al. 2018; Mead et al. 2015; Nehm et al. 2009; Rissler et al. 2014; Scharmann and Harris 1992). Despite widespread recognition of its importance for evolution education research and practice, only a few studies in evolution education have empirically quantified conflict (e.g., Barnes et al. 2020; Konnemann et al. 2018; Nehm and Schonfeld 2007), which is undoubtedly related to the paucity of easy-to-administer assessment tools for this topic (cf. Nehm and Mead 2019). Developing robust measures of conflict with evolutionary ideas could: facilitate more frequent measurement in educational settings, raise instructor awareness of conflict levels in students, and foster rigorous evaluations of educational interventions designed to reduce conflict (e.g., Ha et al. 2015; Nehm et al. 2009; Scharmann and Harris 1992). Such a measure could also lead to conceptual advances in the field of evolution education, as discussed below.

First, feelings of conflict may impact evolution acceptance in ways that have not been precisely accounted for in prior work. For example, religiosity and evolution knowledge are commonly explored contributors to evolution acceptance (Mead et al. 2015; e.g., Bailey et al. 2011; Barone et al. 2014; Dagher and BouJaoude 1997; Deniz et al. 2008; Dunk et al. 2017; Glaze et al. 2014; Heddy and Nadelson 2013; Lombrozo et al. 2008; Paz-y-Minos and Espinosa 2009; 2011; Nehm et al. 2009; Sinclair et al. 1997; Truong et al. 2018). Indeed, some researchers have suggested that religiosity is the greatest predictor of acceptance (e.g., Barnes and Brownell 2017; Glaze et al. 2014; Rissler et al. 2014). However, while it has been shown that individuals may be conflicted about evolution because of their religious beliefs and cultures, they may also be conflicted for many other reasons (and religious respondents may not be conflicted at all). Nehm et al. (2009), for example, found that conflict with evolution had only a modest relationship with religiosity and was more strongly associated with degree of acceptance (Nehm et al. 2009). Additionally, cross-cultural studies in non-religious societies (e.g., China) have found only moderate levels of overall evolution acceptance (Ha et al. 2019). Studies such as these indicate that the relationships between religiosity and acceptance require further scrutiny. This work also suggests that conflict with evolution has the potential to be important as (i) a mediator of the relationship between evolution acceptance and religiosity, and (ii) a unique explanatory factor of acceptance that encompasses a more expansive range of conflict measures beyond those related to religiosity.

Second, broadening the scope of conflict measurement (i.e., perceived family and community conflict with evolution in addition to personal conflict) could reveal contributors to personal conflict as well as their interrelationships. Prior work suggests that the attitudes perceived to be held by members of one’s social groups (e.g., family, friends, teachers, church members) impact personal conflict with–and acceptance of–evolution (e.g., Barnes et al. 2017b; Donnelly et al. 2009; Hill 2014; Winslow et al. 2011). Affinity towards certain social groups (e.g., one’s community) could therefore be an important contributor to one’s personal conflict with evolution (e.g., personal level conflict). Individuals could have *different* causes of personal conflict (e.g., familial, community, religiosity, low evolution knowledge, combinations thereof), which could in turn contribute to different magnitudes of perceived conflict and evolution acceptance. Measuring perceptions of conflict at the family and community scale could therefore help to reveal important and measurable indirect causes of the observed differences in evolution acceptance that have been documented among demographic groups (see Bailey et al. 2011; Metzger et al. 2018; Sbeglia and Nehm 2018). In other words, it is possible that variables such as race or gender could moderate the strength of the relationship between community conflict (or family conflict) and personal conflict. Furthermore, by including multiple hypothesized causes of conflict (many of which may be correlated with each other) into an appropriate model, it would reveal their unique impacts on personal conflict, and could show that religiosity alone has a smaller relationship (direct or indirect) with evolution acceptance than is currently thought. Therefore, the SECM allows researchers to test if a respondent’s social relationships are additional important contributors (direct or indirect) to personal conflict and evolution acceptance. Overall, much more needs to be known about perceptions of personal, familial, and community conflict and their potential interactions with other variables.

In summary, greater understanding of the scales of conflict with evolutionary ideas has potential for advancing conceptual understanding within the evolution education research community and for enhancing educational practices and outcomes relating to evolution (e.g., reducing student conflict magnitudes). The Scale of Evolutionary Conflict Measure (SECM) was developed as a first step towards advancing work in this area. Below we begin with a description of the conceptual framework for measurement, and continue with the corresponding conceptual framework for conflict that guided development of the SECM instrument.

## Conceptual framework: measurement

Evolution education researchers have approached the measurement of latent constructs (like conflict) from different conceptual and methodological perspectives (see Nehm and Mead 2019). Indeed, many types of reliability and validity evidence may be used to support claims about what evolution education instruments are able to measure (Campbell and Nehm 2013; Messick 1995). Validity and reliability evidence for the SECM was gathered in alignment with the *Standards for Educational and Psychological Testing* (AERA et al. 2014); reliability refers to the degree to which instrument measures are replicable, stable, and free from error, and validity refers to the degree to which evidence and theory support the interpretations of test scores for the proposed uses of tests.

Different conceptual frameworks exist for validity. This study adopted a construct validity framework, which encompasses the gathering of evidence in alignment with several separate but interrelated categories (Messick 1995; Campbell and Nehm 2013; AERA et al. 2014), specifically: (i) evidence based on test content (i.e., content validity), evidence based on internal structure (i.e., internal structure validity); (iii) evidence based on relationships to other variables (i.e., convergent and/or discriminant validity); (iv) evidence based on response processes (i.e., substantive validity); (v) validity generalization (i.e., generalization validity); and (vi) evidence of consequences. Many studies are typically needed to capture the full range of evidence needed to establish construct validity. In this study, we investigate content validity, internal structure validity, convergent validity, and substantive validity.

To generate evidence based on test content for the SECM, we used a literature review and expert judgments to specify the content domain, conceptualize the target construct, and operationalize it in the form of closed-response items. Content validity addresses the relevance and representativeness of test content in light of the intended construct (AERA et al. 2014). Evidence for content validity can involve logical or empirical analyses of the extent to which the test content represents the intended content domain (AERA et al. 2014).

To generate evidence based on internal structure for the SECM, we used Rasch modeling (Campbell and Nehm 2013; see Boone et al. 2014 and Boone 2017 for digestible introductions into Rasch modeling). Evidence based on internal structure addresses how well the instrument generates robust measures of the desired latent construct (AERA et al. 2014). A latent construct is a feature that cannot be directly observed (e.g., evolution acceptance, perceptions of conflict, religiosity). In order to generate robust measurement of a latent construct, specific characteristics of the underlying data must be present. These characteristics can differ based on the nature of the response data (e.g., linear, dichotomous, ordinal, etc.) and the modeling approach used, but these criteria must be present for an instrument to be able to generate robust measures of the latent construct (Borsboom et al. 2005). Different modeling approaches are best suited to different types of response data. Rasch analysis, and Item Response Theory (IRT) more broadly, are the most appropriate approaches for estimating continuous latent measures from quantitatively-ordered response data (de Ayala 2019; Hambleton and Jones 1993; Linacre and Wright 1993; Neumann et al. 2011).

To generate evidence based on relationships with other variables, we used correlations and Structural Equation Modeling (SEM) of theoretically associated variables. This form of validity evidence addresses the degree to which measures external to the instrument relate in a theorized manner (AERA et al. 2014). In this way, evidence based on relationships with other variables can support both the conceptualization of the construct and interpretations of the test data (AERA et al. 2014). External variables may include measures that address the same or similar constructs, measures that the test is expected to predict or cause, and group membership variables (e.g., race) that have a theorized relationship with the instrument (AERA et al. 2014; Mueller and Hancock 2019). Simple correlations may provide evidence of associations among theoretically related variables, but evaluating hypothesized causal relationships requires a causal modeling approach such as SEM. SEM allows researchers to evaluate the extent to which the covariances in the instrument-derived data align with (i.e., “fit”) a pre-specified causal hypothesis of how variables should interact (Mueller and Hancock 2019). Therefore SEM is a powerful approach for testing hypothesized causal relationships about latent variables.

## Conceptual framework: conflict perception

Development of the SECM instrument was guided by a conceptual framework for conflict perception, which was in turn supported by a literature review. Our literature review indicated that conflict perception should encompass three core attributes: first, it should consider the hierarchical nature of human social structures (personal, family, community); second, it should probe perceptions of conflict as it relates to variables such as human values, cultures, and beliefs; and third, it should not constrain the operationalization of conflict to single topics or identities (e.g., those related to religion) given that too little is currently known about the diverse array of possible ways that conflict with evolution may manifest itself within respondents (Brem et al. 2003). We discuss the details of these core attributes below.

### Scales of conflict: Personal, family, community

An individual’s perception of conflict with evolution is likely to be situated within broader social contexts, and prior work suggests that the attitudes that are thought to be held by members of one’s social group (e.g., family, friends, teachers, church members) impact personal conflict with evolution (e.g., Barnes et al. 2017b; Donnelly et al. 2009; Hill 2014; Winslow et al. 2011). Barnes et al. (2017b), for example, found that parental attitudes towards evolution were strongly associated with evolution acceptance. Likewise, Winslow et al. (2011) demonstrated that parents, and to a lesser extent church members, were strongly associated with personal views of evolution. Hill (2014) found that adolescent respondents who identified as religiously-devoted creationists were twice as likely to increase acceptance of evolution in early adulthood if they had friend networks in which not all members shared the respondent’s religious ideal type (religious ideal type is a multifaceted construct that includes indicators of religious participation, importance of faith in daily life, feelings of closeness to God, frequency of prayer, etc.). Hill argued that the amount of heterogeneity or homogeneity of religious ideal type within friend networks could act to break down or maintain creationist beliefs (Hill 2014). These studies suggest that the social groups to which individuals belong are related to personal conflict and overall acceptance of evolution.

This prior work motivated the conceptualization of the construct ‘perception of evolutionary conflict’ at multiple, broadly defined social scales, namely: personal, family, and community. These three scales seek to capture the diversity of group memberships that a person may hold. “Family” is intentionally inclusive, although it typically refers to groups that share common ancestry and/or cohabitate, but can extend beyond these groups. Definitions of community in the literature are designed to encompass many different social groupings (e.g., friends, partners, coworkers, online networks, and groups that share common geographies [e.g., neighbors] or common characteristics [e.g., career interest groups, racial or ethnic identities, religious affiliations]). Using open-ended interviews and a diverse group of respondents (n = 118), MacQueen et al. (2001) reported that community was defined similarly by respondents as “a group of people with diverse characteristics who are linked by social ties, share common perspectives, and engage in joint action in geographical locations or settings” (p. 1929). While the associations of family and community with perceptions of conflict and evolution acceptance have been advanced in the literature, researchers have yet to (i) develop measurement instruments that include such relationships or (ii) evaluate them in a causal modeling framework. The SECM was designed to allow the formal testing of hypotheses related to multiple scales of conflict.

### Operationalizing perceptions of conflict: belief, culture, values

For each scale (personal, family, community), the perception of conflict with evolution was conceptualized as a construct that can be operationalized using several interrelated but distinct variables. Three variables from prior work (e.g., Barnes et al. 2017a, b; Brem et al. 2003; Dagher and BouJaoude 1997) that may be used to operationalize this construct include: (i) the level of conflict with one’s beliefs, (ii) the level of conflict with one’s culture, and (iii) the level of conflict with one’s values. It is important to note that religious identities may encompass all three of these variables; however, beliefs, culture, and values may also operate outside of a religious context and may be connected to broader aspects of identity. While relatively little work has focused on the non-religious aspects of beliefs, culture, and values as they relate to evolution acceptance (Brem et al. 2003 is an important exception), substantial work has been carried out on these elements (and their connections to identity) more broadly. Below we provide a brief review of each of these variables.

Beliefs are considered to be a way of knowing derived from personal truths, as opposed to world truths (Smith et al. 1995). Beliefs tend to be highly subjective, firmly structured, and unaffected by empirical evidence (to the extent they are confronted with it) (Smith et al., 1995; Southerland et al. 2001). Students hold beliefs about many topics, but religious beliefs in particular have been suggested to be strongly associated with perceptions of evolutionary conflict (Barnes et al. 2017a, 2017b; Rissler et al. 2014; Truong et al. 2018). Religious beliefs refer to the “specific beliefs one holds about the existence and influence of a deity” (Barnes and Brownell 2017, p. 3). Although evolution is often presented as incompatible with religious beliefs (e.g., Coyne 2015; Dawkins 2009), authors have shown that interventions designed to highlight their compatibility were associated with a reduction in the perception of conflict (e.g., Barnes and Brownell 2017; Nehm and Schonfeld 2007; Truong et al. 2018).

Culture encompasses the values, assumptions, practices, and artifacts that are shared within a group, community, or society (Taras et al. 2009). Culture becomes part of an individual through consistent engagement with family and community members (Causadias et al. 2018). Thus, culture operates at both the individual and group level in the sense that it implies belonging to a social entity that conditions one’s experiences (Taras et al. 2009). There are many models of culture that emphasize different elements of this construct. For example, Gelfand et al. (2006) advanced a model of culture that is based on the notion of cultural looseness vs. tightness, which refers to the degree to which social norms are enforced within the group. Leung et al. (2002) offered a model that describes cultures by their basic assumptions about social complexity, spirituality, perceived fate control, cynicism, and rewards. A person’s culture may bear on perceptions of conflict with evolution if evolutionary theory is viewed as incompatible with shared assumptions or norms within their group, especially if a particular group enforces strict adherence to these norms. In particular, evolutionary ideas have been perceived to be at odds with one’s religious culture (defined as “the sociocultural norms that individuals experience related to religion.” [Barnes and Brownell 2017 p. 38]) and this sense of religious cultural conflict need not require the perception of a contradiction with one’s religious beliefs (Barnes and Brownell 2017). For example, cultural tightness related to the literal vs. non-literal interpretations of religious texts could explain the association between individuals’ perceptions of conflict with evolution and their specific religious affiliation (Dagher and BouJaoude 1997). Interventions designed to highlight the compatibility between evolutionary ideas and the perceptions of leaders of religious communities have been shown to be associated with an increase in acceptance (e.g., Manwaring et al. 2015). Other groups may also experience conflict related to their group memberships. For example, ethnicity and race are intimately related to, or are a part of the broader concept of culture (Causadias et al. 2018), and some race groups have been found to have lower levels of evolution acceptance than others (Bailey et al. 2011; Metzger et al. 2018; Sbeglia and Nehm 2018). Unfortunately, few studies disaggregate evolution acceptance by race (Mead et al. 2015) and it is not currently known how the documented differences in acceptance relate to the magnitude or nature of perceived conflict with evolution. Nevertheless, although culture is known to be an important factor shaping perceptions, the assumption that the role of culture is stronger for minority than majority groups has been challenged (e.g., Causadias et al. 2018) and investigations seeking to explain differences in acceptance should proceed with caution.

Values refer to the ideals that are central to one’s personhood and identity (Hitlin 2003). They are a set of concepts or beliefs about desirable end states or behaviors that tend to have several important features: a) they are trans-situational (i.e., context-independent) and often immutable over time, b) they guide the selection of behaviors and the evaluation of events, and c) they are (or can be) well-organized mental structures that are ordered by their relative importance (Hitlin and Piliavin 2004; Michener et al. 2004; Schwartz and Bilsky 1987). Therefore, values parameterize perceptions of acceptable or ethical behaviors and events, structure interpretations of personal experiences, and orient people to their social context (Hitlin and Piliavin 2004; Marini 2000). While ideologies (religious or otherwise) can overlap with and inform values (Maio et al. 2003), values extend beyond individual contexts (e.g., religious or cultural contexts) (Hitlin and Piliavin 2004) and more broadly structure views about the world. Schwartz (1992; 1994) has empirically evaluated and outlined a structure of ten human values that he argues are near-universal. These include universalism (“tolerance and concern for welfare of all others”), benevolence (“preserve and enhance welfare of those with whom one is in frequent personal contact”), and self-direction (“autonomous thought and action [idea of agency]”). These values are well-aligned with some of the negative perceived societal implications of evolutionary theory outlined and observed by Brem et al. (2003), including the naive perception that evolution implies a lack of control or self-determination and justifies selfishness and racial or ethnic discrimination. In one study, for example, the majority (56% and 65%, respectively) of college students reported that accepting evolutionary ideas makes it easier to justify racism and ethnic discrimination and harder to think of people as determining their own fate (Brem et al. 2003). In summary, the literature supports the roles of beliefs, cultures, and values in the conceptualization of perceptions of conflict with evolution.

## Research questions

In this paper, we aim to evaluate if the SECM instrument productively measures the intended construct. Specifically, we ask:

1. Does the SECM adhere to well-accepted criteria of robust measurement? (1.1) Do the items that comprise the instrument display acceptable fit to model expectations? (1.2) Is the instrument best modeled as one dimension or three dimensions? (1.3) Does the instrument reliably order items by their agreeability, and respondents by their measures on the latent trait? (1.4) How precisely does the instrument measure the latent trait? (1.5) Does the rating scale function as expected? (1.6) Does the instrument display measurement invariance pre- and post-instruction?
2. Are respondents interpreting the items as anticipated?
3. Are latent SECM measures convergent with measures of similar constructs?
4. Do measures of conflict derived from the SECM contribute to the explanation of evolution acceptance above and beyond the contributions of religiosity and evolution knowledge?

## Materials

### Participant sample

Participants were drawn from two semesters (Fall 2019 and Spring 2020) of an introductory biology course at a large, public, research-oriented university in the northeastern United States (N = 1179 for the pre-test [~ 90% participation rate]). All students were enrolled in introductory biology courses in which evolution was a major theme (nearly all units connected to evolution in some way). Participants were asked to self report background characteristics including age, gender, ethnicity or racial identity (White, Asian, or underrepresented minority [URM, including Black/African American, American Indian/Alaska Native, Hispanic of any race, Native Hawaiian/Other Pacific Island]), whether English was their first language, and self-rated reading and writing ability (as an indication of English language proficiency—scale ranges from very poor to excellent). Academic information collected included undergraduate class standing (freshman, sophomore, junior, senior), plan (biology, Non-Bio STEM [science, technology, engineering, or mathematics], non-STEM), and prior biology coursework. Participant demographic and background information is summarized in Table 1. The sample of students included both majors and non-majors and had representation from diverse backgrounds (in terms of race, ethnicity, and gender [see Table 1]). The sample was also chosen because evolution was a core idea in the courses, and accordingly was anticipated to spur thinking about evolution. In both semesters, the nature of science and evolution instruction occurred within the first few weeks of the semester. The pre-survey took place during the nature of science unit but before the evolution unit.

**Table 1:** Sample size, participation rate, and background information

	Fall 2019	Spring 2020
Sample size		
SECM	444 pre and post^a^	728 pre
I-SEA, CANS, Religiosity, IOS	444 pre	728 pre
Participation rate	91%	89%
Background variables		
Race		
% Asian	50%	47%
% URM	23%	20%
% White	27%	32%
% female	52%	59%
% non-Bio major	40%	41%
% ELL	29%	28%
% no prior bio	31%	27%
% freshman or sophomores	49%	34%
% poor reading ability	1%	1%
% poor writing ability	1%	1%

^a^The same students took the pre- and post-survey, but the post was only used for DIF analysis

### Instrument development

A literature review as well as faculty experts from two fields (evolution education and social psychology) were used to conceptualize a three-scale construct (i.e., perceptions of personal, family, and community conflict), each of which was operationalized using three closed-response items designed to capture perceptions of conflict at each scale between evolutionary ideas and (i) values, (ii) culture, and (iii) beliefs (n = 9 items total). Specifically, each of the nine items of the SECM had the following structure:

**Stem:** Evolutionary ideas are at odds or in conflict with…

**Scale:** …[my, my family’s, my community’s]…

**Variable:** …[culture, values, beliefs].

**Fig. 1:**
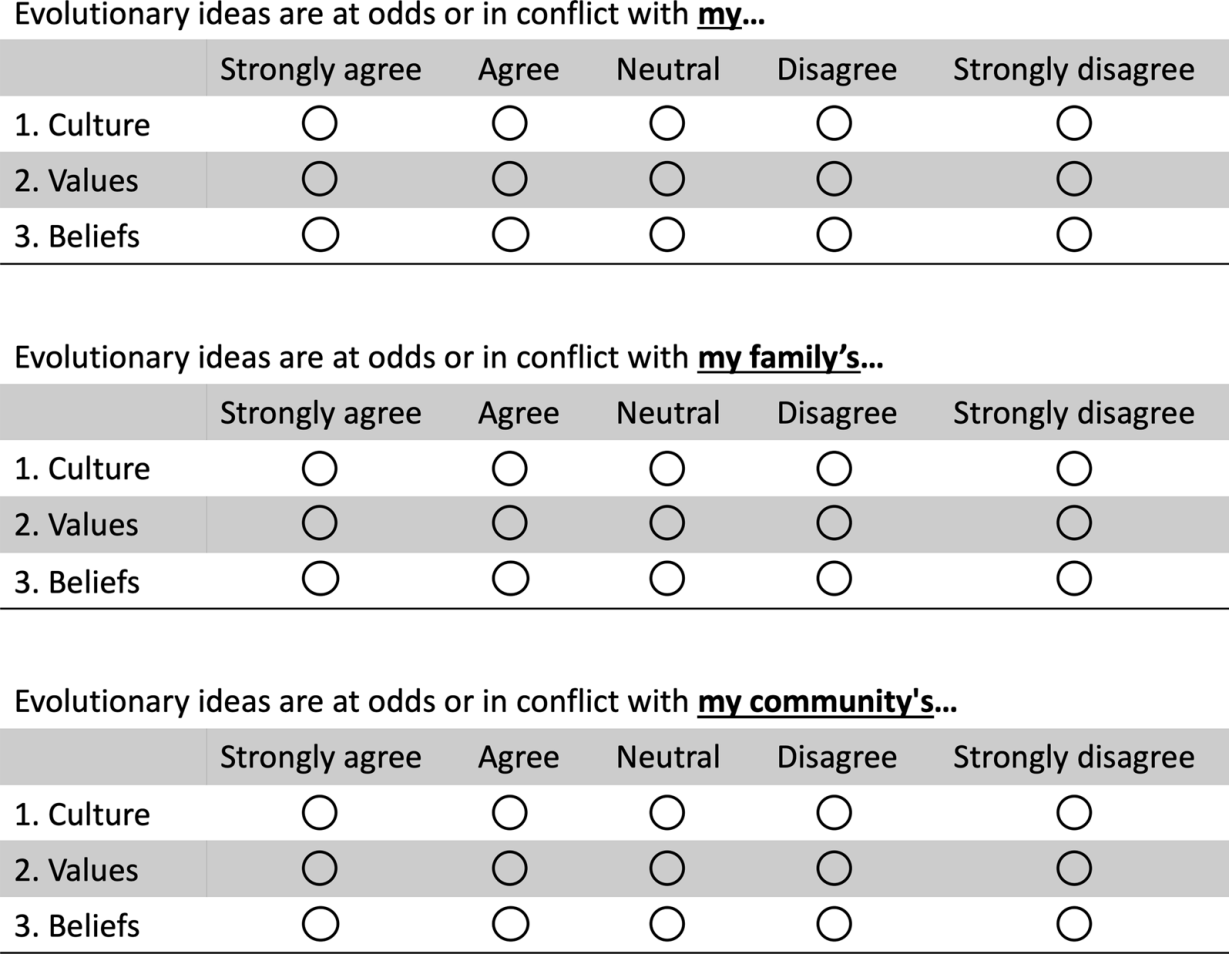
Text and recommended administration format for the SECM

The stem was derived from Nehm and Schonfeld (2007)’s conflict measure and is common to all items; every combination of scale and variable was designed to compose the full 3-scale instrument. The items have a five-option response format (strongly agree [SA], agree [A], neutral [N], disagree [D], and strongly disagree [SD]). Responses were coded from 0–4, with 4 representing the highest perceived conflict. See the full instrument in Fig. 1.

### Administration and data sources

We administered a survey to students at the beginning and end of the semester in Fall 2019 and the beginning of the semester in Spring 2020. The survey included instruments that measure perceptions of evolution conflict (SECM), evolution acceptance (Inventory of Student Evolution Acceptance [I-SEA], Nadelson and Southerland, 2012), evolution knowledge (Conceptual Assessment of Natural Selection [CANS], Kalinowski et al. 2016), religiosity (Cohen et al. 2008), and compatibility perception (Inclusion of Others in Self [IOS] measure, Aron et al. 1992). After collecting response data, we evaluated the reliability, item fit, and dimensionality of each instrument using Rasch analysis (see methods section for details on Rasch analysis). There were no missing data for any of the instruments, but 21 respondents had missing background data and were excluded from relevant analyses. We describe the I-SEA, CANS, IOS, and religiosity instruments below.

#### I-SEA

The I‐SEA measures evolution acceptance (Nadelson and Southerland 2012). It contains three item sets (eight items each, 24 total), each representing a different evolutionary scale or taxon: microevolution, macroevolution, and human evolution. The scale and taxonomic differences among item sets may be considered surface features that are irrelevant to expert-like evolutionary reasoning (Nehm and Ha 2011). The items have a five‐option response format (strongly disagree [SD], disagree [D], undecided [U], agree [A], and strongly agree [SA]). Responses were coded from 0–4, with 4 representing the highest evolution acceptance. Items with negative valences were reverse-coded as appropriate. When necessary, adjacent categories were collapsed for those items in which one or more categories lacked responses. Existing validity evidence includes content validity (e.g., student and expert interviews, Nadelson and Southerland, 2012), and internal structure validity evidence (e.g., Rasch-based fit statistics, reliabilities, item functioning, dimensionality analysis, pre-post instruction changes; Sbeglia and Nehm 2019).

#### CANS

The CANS measures knowledge of natural selection (Kalinowski et al. 2016). The instrument contains 24 multiple choice items presented in clusters that focus on specific taxa: anteaters, whales, cacti, mosquitos. The instrument’s authors chose to organize the instrument by taxon to allow students to reason across biological contexts, and to address misconceptions best suited to particular cases (e.g., role of use and disuse, evolution in plants vs. animals, evolution in relation to human disease; see Nehm et al. 2012). Some of the item clusters contain items that are parallel in form but differ in taxon. The items have one correct answer and were coded such that incorrect responses were recorded as a “0” and correct responses as a “1”. Existing validity evidence includes content validity (e.g., student interviews and expert reviews) and internal structure validity (e.g., IRT-based fit statistics, reliabilities, pre-post instruction changes) (Kalinowski et al. 2016).

#### Religiosity

The religiosity instrument was developed by Cohen et al. (2008). The instrument contains nine items, seven of which ask about the respondent’s religious identity, and two of which ask about the respondent’s religious participation. The items have a five‐option response format (i.e., strongly disagree, disagree, neutral, agree, and strongly agree). Responses were coded from 0–4, with 4 representing the highest religiosity. Existing validity evidence includes internal structure validity (e.g., reliability) and convergent validity (Cohen et al. 2008).

#### IOS

**Fig. 2:**
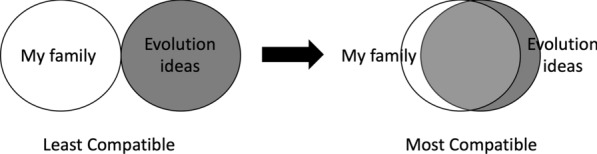
Example answer options of the modified IOS item to measure respondents' perceived compatibility between their family and evolution ideas. Survey respondents selected one of 7 pairs of overlapping circles

The IOS measure is designed to evaluate how closely (or compatible) respondents felt to another person or group (Aron et al. 1992). In the original conceptualization of the instrument, respondents are presented with seven pairs of circles that varied in degree of overlap. One circle in each pair would be labeled “self,” and the second circle was labeled as some other group. The instrument has been adapted to study a variety of domains (e.g., Aron et al. 1992; Clark et al. 2016; Shin et al. 2016; Tropp and Wright 2001). Existing validity evidence for this instrument includes internal structure validity (e.g., alternate-form and test–retest reliability), convergent validity, and external structure validity (e.g., relationships with other variables) (Aron et al. 1992). We modified the instrument to measure respondents' perceived compatibility between their family and evolution ideas (Fig. 2). Respondents in our sample were asked, “Which of the 7 pictures below best describes how compatible you think your family is with evolutionary ideas and concepts?”.

## Methods

### RQ1: Does the SECM adhere to well-accepted criteria of robust measurement?

To address RQ1, we modeled each scale using an approach that is appropriate for the type of response data (i.e., ordered) and the structure of the latent construct (i.e., continuous). For each of these considerations, Rasch analysis is appropriate (de Ayala 2019; Liu 2010). Specifically, we modeled the SECM using a partial credit Rasch model (i.e., item + item*step, PCM2 in TAM) with the R package Test Analysis Modules (TAM, v. 2.10–24, Robitzsch et al. 2018). Rasch analysis, and IRT more generally, estimates respondents' latent measures using a probabilistic approach, and thus does not claim to measure a true score. Rather, a respondent’s likelihood of selecting a particular response is based on the difference between a respondent’s measure on the trait and each item’s level of agreeability (or difficulty). These approaches theorize that in order to generate robust measures of a latent construct, the following characteristics of the underlying data must be present: (1) acceptable item fit, (2) acceptable rating scale functioning, (3) unidimensionality, (4) acceptable item and person reliability, (5) acceptable person-item alignment (Wright maps), and (6) measurement invariance (Boone 2017; Boone et al. 2014). These six criteria may be considered a benchmark for productive measurement using the Rasch model, and if met, suggest that the instrument can generate robust measures of the latent construct (Borsboom et al. 2005). Here, “measures” refers to both an item measure (i.e., the agreeability or difficulty of an item) and a person measure (i.e., the agreeability or ability of a person). Item and person measures are on the same logit scale and can be compared to each other (Boone et al. 2014). In Rasch analysis, unlike IRT, the item measure is the only parameter considered in the calculation of the person measure (using a weighted maximum likelihood estimation [WLE] of the item parameter). IRT models, on the other hand, also include other parameters that can be added or removed to improve the fit of the model. Rasch analysis assumes that no additional parameters are needed for productive measurement of a latent construct (Boone et al. 2014). Therefore, although Rasch analysis and IRT are considered to be conceptually different approaches, the Rasch model is mathematically equivalent to a 1-parameter (1PL) IRT model (Boone et al. 2014). A benefit of the strict 1-parameter assumption of the Rasch model is that it calibrates instruments using an equivalent standard (Romine et al. 2017); the probability of selecting a particular level of conflict for an item is proportional only to the difference between the agreeability of the item and the level of conflict of the respondent. Furthermore, this approach converts raw, ordered data to a continuous linear scale, making Rasch and IRT measures suitable for parametric statistical analyses. We briefly summarize each of these evaluation criteria below.

#### Item fit

To address if the items that compose the instrument have an acceptable fit to model expectations (RQ 1.1), we analyzed the information-weighted (i.e., Infit) and unweighted (i.e., Outfit, which is sensitive to outliers) mean squares fit (MNSQ) statistics for each item. In alignment with psychometric standards, we considered MNSQ fit values of 0.5–1.5 logits to be acceptable (Boone et al. 2014). Fit values that were slightly outside this range indicate that an item does not meaningfully contribute to measurement, and values > 2 indicate that the item is degrading to measurement (Boone et al. 2014).

#### Rating scale functioning

To evaluate if the rating scale of the SECM functions as expected (RQ1.5), we used two approaches. First, we examined the correspondence between the participants’ answer choices and their overall Rasch person measures (Boone et al. 2014; Sbeglia and Nehm 2018, 2019). Well-functioning items should have a high correspondence. In the second approach, we examined the Rasch-Andrich thresholds (also called step parameters or Andrich deltas), which represent the locations on the Rasch category probability curve where the curves for adjacent answer options meet, and indicate the point at which there is a 50% probability of selecting adjacent answer categories (Linacre 1999). Thresholds that are close together, or not in the expected sequential order (e.g., “strongly agree”, “disagree, agree”), are said to be disordered. Depending upon the cause of the anomaly, threshold disorder may or may not indicate that the item is unable to predictably discriminate abilities on the latent trait (Adams et al. 2012; Andrich 2013; Boone et al. 2014). Collectively, we used rating scale functioning and item fit to assess the overall functioning and appropriateness of each item in the SECM.

#### Dimensionality

The items of an instrument must measure only one construct or topic (i.e. be unidimensional) in order for the resulting latent measures to indicate the relative position of respondents along the same trait. Therefore, it is necessary to evaluate the dimensionality of the item sets. We conducted two analyses to determine if the instrument is best modeled as one dimension (all conflict scales combined) or three dimensions (each scale on a separate dimension) (RQ1.2). First, we used a principal components analysis (PCA) of the residuals from a unidimensional Rasch model to evaluate patterns of unexplained variance. If the eigenvalue of the first contrast is greater than 2, it indicates sufficient unexplained variation among the residuals to suggest the possibility of additional, unmodeled dimensions (Boone et al. 2014). We also plotted the eigenvalue of the first PCA contrast against the agreeability of each item to visualize the pattern of shared unexplained variation among items. Items that cluster together can be hypothesized to represent a distinct dimension. This approach allows additional dimensions to be discovered based on patterns of unexplained variation.

Second, we used a likelihood ratio test to compare the relative fit of unidimensional and multidimensional models of the response data to Rasch expectations (see Robitzsch et al. 2018). In this approach, dimensions are hypothesized a priori and the resulting models are tested for data-model fit.

#### Item and person reliability

Item reliability quantifies the extent to which the instrument is able to consistently order items by their difficulties, and may be measured using expected a posteriori/plausible value reliability values (EAP/PV) (Bond and Fox 2001). Person reliability quantifies the extent to which an instrument is able to order respondents based on their abilities, which can be measured using Warm's Mean Weighted Likelihood Estimates (WLE) (Bond and Fox 2001). Reliabilities range from 0 to 1 and can be interpreted much like Cronbach’s alpha (Boone et al. 2017). Values > 0.70 are acceptable (Grigg and Manderson 2016; Yang et al. 2017). Collectively, these measures indicate the ability of the instrument to reliably order items by their agreeability and respondents by their level on the latent trait (RQ1.3).

#### Person-Item alignment

The alignment of an instrument to the sample in which it was administered indicates the level of measurement precision the instrument can achieve. Precise measurement occurs when the agreeability of items or of the categories on the rating scale (for polytomous items) span the full spectrum of respondent abilities, and precision declines when the items and respondents are less aligned. Items or categories that differ in agreeability act like tick marks on a ruler that allow you to bin respondents based on their abilities. The fewer distinct tick marks on the ruler, the fewer bins respondents can populate, and the lower the precision of measurement. To measure how precisely the SECM measures the latent trait (RQ1.4), we visualized person-item alignment using Wright maps. Wright maps plot Rasch item difficulties against Rasch person measures. If the instrument is polytomous (i.e., not dichotomous) in nature, Thurstonian thresholds for each rating scale category may also be plotted for each item. Thurstonian thresholds are the locations on the Wright map where a respondent has a 50% probability of selecting a particular answer category (or higher) for an item. For this format of instrument, item agreeability is the mean of the Thurstonian thresholds (see Sbeglia and Nehm 2019 for more detail). Respondents with high abilities on the latent trait are positioned at the top of the Wright map. Likewise, items and thresholds with the highest agreeabilities are also positioned at the top of the map, which reflects their high agreeability because top students only have a 50% probability of choosing a given answer (less able students have lower probabilities).

#### Measurement invariance

Measurement invariance describes situations in which the underlying measurement structure of an instrument (e.g., item descrimination/factor loadings, item thresholds, residual variances, dimensionality) remains stable through time (or across groups) (van de Schoot et al. 2015). While test respondents are often expected to show a change in their *amount* of a particular latent trait through time (e.g., knowledge of evolution before and after taking a biology course), the underlying measurement structure of the instrument must remain stable in order for a comparison of latent measures to be meaningful (Lommen et al. 2014). To establish if the SECM displayed measurement invariance pre- to post-instruction (RQ1.6), we conducted a differential item functioning (DIF) analysis on the SECM items. An item displays DIF when respondents with equal abilities, but from different groups or time points, differ in their expected responses for the item. An item has “non-DIF” if respondents with equal abilities have the same expected response, regardless of group or time. A finding of “non-DIF” from the pre- to the post-survey would suggest measurement invariance, and thus allow for the meaningful comparison of SECM measures across time. DIF may be calculated by running a multifaceted Rasch model in which the variable being examined (the facet, in this case time) is modeled as having an interaction with each item (Robitzsch et al. 2018). To evaluate the significance of DIF, the absolute value of the t-ratio for the interaction parameter must be greater than 2. If the SECM does not exhibit DIF from pre-to post-course, it may be considered to have measurement invariance, and therefore pre-post comparisons can be meaningfully made.

### RQ2. Are respondents interpreting items as anticipated?

In order to gather evidence to test the claim that respondents were interpreting SECM items as anticipated (i.e., substantive validity evidence), a sample of 619 students completing the SECM were also asked to answer a follow-up question. This question was used to examine the correspondence between the intended interpretation of the “community” item and participants’ actual definitions of community. After answering the “community” item, respondents were asked to select the specific groups that they considered to be part of their community. Each respondent was allowed to choose and rank a maximum of three of the following options, or no option at all: (1) My friends at college, (2) My friends from high school, (3) My significant other or partner, (4) People in my major or professional track, (5) People from my race group, (6) People from my neighborhood, (7) People from my church or who share my religion, (8) People from my place of work, and (9) People from my online social network. The first choice was indicated as the choice most important to one’s community.

We performed two analyses. First, we analyzed the correspondence of our intended interpretation of the community item (see above) and participants’ actual chosen definitions by evaluating the proportion of the sample that selected “Not applicable” for one or more of the three specific community categories. This response was interpreted as indicating that the categories of community defined in our conceptual framework and offered to students were not well-matched to their definition of community. Second, we analyzed if respondents defined their communities similarly to one another by evaluating if a subset of categories were more frequently selected than others, and if this pattern differed by conflict level. A 2-sample z-test was used to test for the equality of proportions between high and low conflict respondents. For this analysis, respondents were separated into high and low conflict categories based on whether their Rasch measures were above or below the population’s mean conflict level. Overall, these analyses on a large sample were used to test the claim that respondents were interpreting the item as anticipated and that respondents from different conflict groups were interpreting the features of the items as designed. We use a critical p-value of 0.01 for all analyses.

### RQ3: Are latent SECM measures convergent with measures of similar constructs?

To address RQ3, we correlated latent measures of each respondent’s perception of their family’s conflict with evolution ideas (i.e., SECM Family item set) with the modified IOS item using a Spearman correlation. As described above, the modified IOS item asked about perceived compatibility between respondents’ families and their evolutionary ideas.

### RQ4: Does the SECM contribute to the explanation of evolution acceptance above and beyond the contributions of religiosity and evolution knowledge?

To address RQ4, we shifted our approach from a Rasch framework to a Structural Equation Modeling (SEM) framework. Whereas Rasch or IRT is a preferred approach when the test and its categorical items are the focus of study (Wright 1996), Latent variable path analysis (LVPA, a SEM method) is preferred when modeling putative causal relationships among latent variables (Mueller and Hancock 2019). LVPA models include a measurement component and a structural (i.e., theoretical) component. The measurement component of a LVPA is akin to a confirmatory factor analysis (CFA), which models latent traits based on the patterns of covariation among its items (i.e., measured variables). CFA and IRT are similar in this regard (though modeling assumptions may differ). However, CFA fits within a broader path analysis framework, in which the measurement model is situated within a structural model of causal relationships among variables. Though CFA and LVPA are traditionally reserved for traits with continuous items (not Likert scale items as in the SECM) due to the use of maximum likelihood estimation (Wright 1996), recent work has resulted in the development of more flexible estimation approaches, including those appropriate for ordered categorical data (e.g., diagonally weighted least squares [DWLS] and its robust variants [e.g., WLSMV]) (Rosseel 2020).

SEM allows the testing of a priori theory-driven hypotheses, and is not designed to generate hypotheses post-hoc (or to model hypotheses derived from previous exploration of the same data set) (Mueller and Hancock 2019). Therefore, the theoretical framework underlying the model being tested must be articulated and justified, which we do in the following section (see section titled *Theoretical framework for SECM factor and item relationships*). Using this theoretical framework, which seeks to outline how SECM factors and items may relate to each other, we built a structural model using LVPA in the R program Lavaan v. 0.6-6 (Rosseel 2020a). However, this particular theoretical framework need not be adopted in order to use the SECM, and we encourage continued discussion on the appropriateness of our proposed relationships.

#### Theoretical framework for SECM factor and item relationships

Individuals who experience personal conflict with normative scientific ideas do so because of the ways in which they perceive or process relevant information and events. These perceptions (along with perceptions more generally) may be linked to a person’s group memberships and resulting social identities (Xiao et al. 2016; Kahan et al. 2007). For example, individuals who identify themselves as being members of a particular group may align their perceptions and perspectives with those of the group (Kahan et al. 2007), which is a phenomenon that has been explicitly connected to evolution acceptance, evolution rejection, and science denial more broadly (Walker et al. 2017,1). Furthermore, exposures to social groups during human development are thought to calibrate peoples' perceptual systems (Xiao et al. 2016), possibly forming cognitive models that can be broadly applied across contexts. Therefore, we propose that aspects of social identity (e.g., the ideas and perspectives held by the social group with which one identifies) may have a causal relationship with one’s personal perceptions of conflict with evolution. Other aspects of identity (e.g., one’s values, cultures, and beliefs) may be indicative of (i.e. manifestations of) one’s latent level of perceived conflict with evolution.

#### Description of the measurement model

Before implementing a structural model that aligns with the theoretical framework for SECM factor and item relationships (described above), we first evaluated the fit of the measurement model. The measurement model is the part of the model that relates the items (i.e., measured variables) with the factors (i.e., latent variables). A well-fitting measurement model establishes that each factor and its associated items acceptably measures the intended construct. Once a well-fitting measurement model is established, hypothesized causal paths among factors may be modeled and evaluated. In a measurement model, factors are linked to their associated items and all factors (or their residuals [i.e., disturbances] if the factors are endogenous) are allowed to covary with each other (Mueller and Hancock 2019). Next, theory should be used to model covariances between the residual variance (i.e., error variance) of appropriate items.  Error variance is the part of the measured variable that does not relate to the factor. If two items have something in common that is not captured by the factor, then their error variances may be correlated with each other (Rosseel 2020b). In order for the measurement model to fit the underlying data, possible error covariances among the items must be considered a priori using theory, and then modeled. Below we detail how we modeled each latent trait in the measurement model.

In alignment with the conceptual framework for conflict perception (see introduction) and the theoretical framework for SECM factor and item relationships (see methods above), the SECM was modeled as three factors, one for each scale of conflict. For each factor, the items (i.e., the culture, values, and belief items) were modeled as indicators (i.e., a reflective relationship between the latent trait and the measured variables [see Mikulić and Ryan 2018 for more on reflective vs. formative models]). Error covariances were modeled among items from different SECM factors that had parallel forms (e.g., the error variances of the three items about “values” were allowed to covary). The CANS was modeled as one factor and error covariances were modeled among items with parallel forms, and among items that focused on the same taxon. Taxon is a feature of instrument items that has been hypothesized to impact evolutionary reasoning and test performance (Kalinowski et al. 2016; Opfer et al. 2012). The I-SEA was modeled as three factors (microevolution, macroevolution, and human evolution) as recommended by the instrument’s authors (Nadelson and Southerland, 2012), and error covariances were modeled among items with negative valence, among items about human microevolution, and among items about human macroevolution, all of which have been hypothesized as possible additional dimensions within the instrument (see Sbeglia and Nehm 2019). Religiosity was modeled as one factor and error covariance was modeled between the two religious participation items. Background variables (i.e., plan, prior biology coursework, level, ELL status, reading and writing ability, gender, race) were also included in this model. All factors were allowed to covary. Modification indices were run and evaluated for possible theory-based changes to the model. We used the WLSMV estimator, which allowed all indicators to be modeled as ordered. Given an acceptable data-model fit for the measurement model, the structural portion of the model could then be estimated (van Riper and Kyle 2014).

#### Description of the structural model

Structural models are built from measurement models, but in structural models, only theoretically important paths are retained. Theoretically important paths are those that align with the theoretical framework for factor and item relationships laid out by the researcher. Specifically, in line with our theoretical framework for SECM factor and item relationships, we built a LVPA model with the following features: The latent traits of family and community conflict perception were modeled as being causal to personal conflict perception, and personal conflict perception was modeled as causal to the three scales of evolution acceptance. Family and community conflict were allowed to covary and the three factors of evolution acceptance were allowed to covary. Background variables (i.e., plan, prior biology coursework, level, ELL status, reading and writing ability, gender, race), evolution knowledge, and religiosity were modeled as having structural paths to all factors within the model, which removes the linear effects of these variables on parameter estimates (i.e., it controls for them) (Mueller and Hancock 2019). This model is visualized in the results section. With these controls in place, we estimated the significance of the causal paths among the scales of conflict, and between the personal conflict and the scales of evolution acceptance by generating asymptotic standard errors of parameter estimates using the Delta method (Rosseel 2020b). This analysis allowed the investigation of the unique contribution of the causal paths between the SECM and evolution acceptance, above and beyond religiosity and evolution knowledge (RQ3).

#### Fit statistics

We used the following fit statistics and cutoffs: root mean square error of approximation (RMSEA) < 0.05, standardized root mean square residual (SRMR) < 0.08, and Comparative Fit Index (CFI) > 0.95 (Mueller and Hancock 2019). If a model has acceptable fit, then the parameters are considered interpretable.

## Results

The mean of raw SECM scores was 1.99/6 (sd = 2.09) for personal conflict, 2.60/6 (sd = 2.42) for family conflict, and 2.51/6 (sd = 2.16) for community conflict. The three faculty experts in evolution education and social psychology agreed that the items in the SECM were clearly worded and appropriately connected to and representative of the construct and the existing literature. The mean raw score of the I-SEA was 26.57/32 (sd = 4.5) for microevolution, 25.62/32 (sd = 4.24) for macroevolution, and 24.69/32 (sd = 5.52) for human evolution. The mean raw score for the CANS was 10.69/24 (sd = 4.68), and religiosity was 15.97/36 (sd = 10.18). Below we answer each of our research questions about the SECM. See Additional file 1: Table S1 for a summary of the psychometric properties of the I-SEA, CANS, and religiosity instrument.

### RQ1: Does the SECM adhere to well-accepted criteria of robust measurement?

**Fig. 3:**
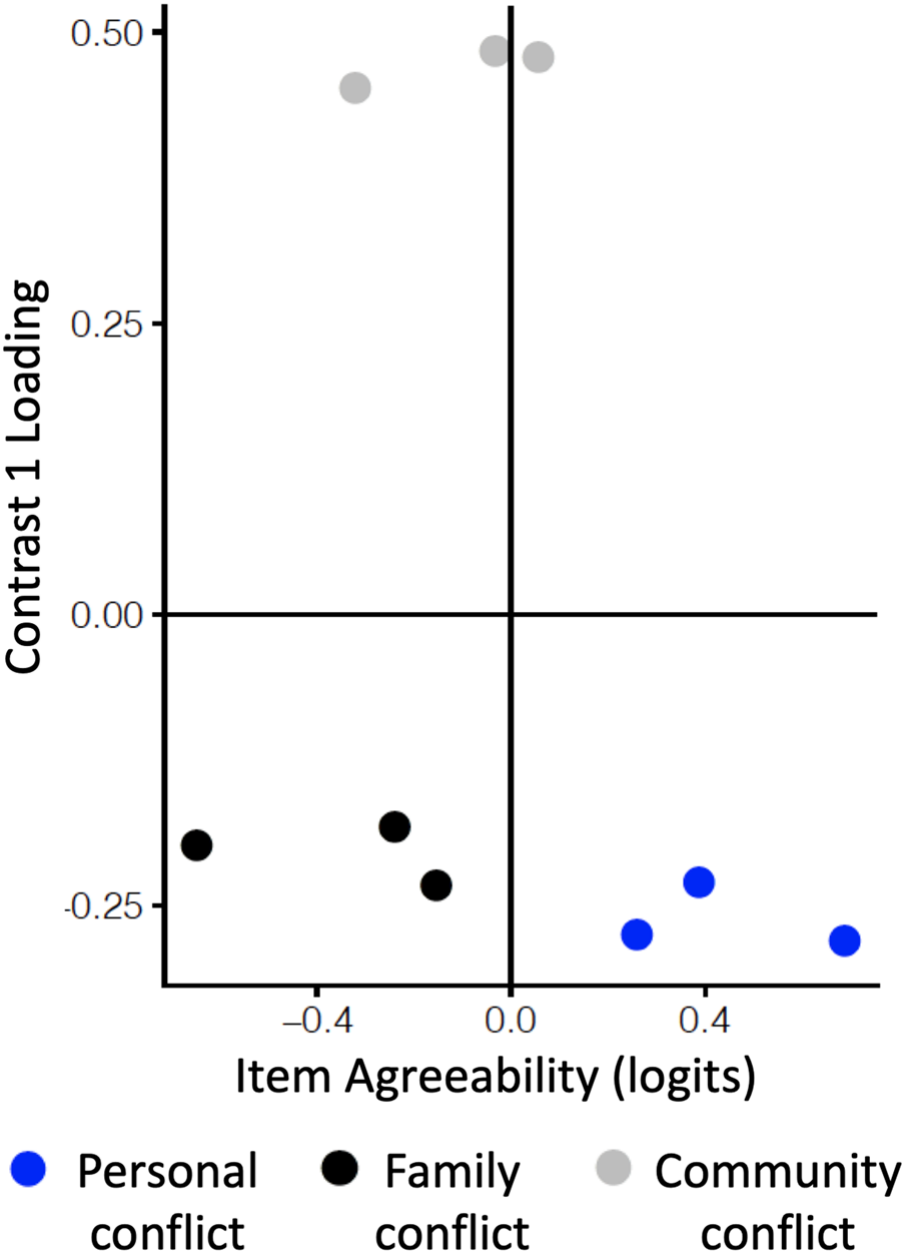
PCA of Rasch residuals from a one-dimensional model

We used responses on the SECM to model evolutionary conflict perception as a one-dimensional and as a three-dimensional construct (i.e., a separate construct for personal conflict, family conflict, and community conflict). For both construct formulations, Rasch fit statistics indicated that the items were generally productive for measurement and no items were degrading to measurement. A PCA of Rasch residuals generated by the one-dimensional model indicated substantial unexplained variation (eigenvalue of the first contrast = 3.33) and clustered items according to the scale of conflict (i.e., personal, family, community), not according to the variable (i.e., values, culture, beliefs) (Fig. 3). A likelihood ratio test confirmed that a three-dimensional model (in which each scale of conflict was modeled as its own construct) was a significantly better fit to the data than a one-dimensional model (*X*^*2*^ = 2578.54, df = 5, p < 0.001; AIC_1D_ = 21,356.93, AIC_3D_ = 18,788.39; BIC_1D_ = 21,544.57, BIC_3D_ = 19,001.39). When modeled as three separate one-dimensional models, the item fit was acceptable and productive for all items (Table 2), the PCA of Rasch residuals indicated little unexplained variation (eigenvalue of the first contrast = 1.75–1.8).

**Table 2:** Item agreeability (mean of the Thurstonian thresholds) and fit statistics for each dimension (i.e., personal conflict, family conflict, community conflict) of the SECM

Item	Mean of Thurstonian Thresholds^a^	Outfit	Infit
Personal conflict 01 (culture)	− 0.13	0.91	1.24
Personal conflict 02 (values)	0.52	0.53	0.77
Personal conflict 03 (beliefs)	− 0.39	0.74	1.07
Family conflict 01 (culture)	0.24	0.91	1.12
Family conflict 02 (values)	0.43	0.74	0.95
Family conflict 03 (beliefs)	− 0.67	1.21	1.44
Community conflict 01 (culture)	0.18	0.65	0.87
Community conflict 02 (values)	0.43	0.61	0.85
Community conflict 03 (beliefs)	− 0.62	0.94	1.20

^a^More negative mean thresholds are easier items to endorse and more positive mean thresholds are more difficult items to endorse

**Fig. 4:**
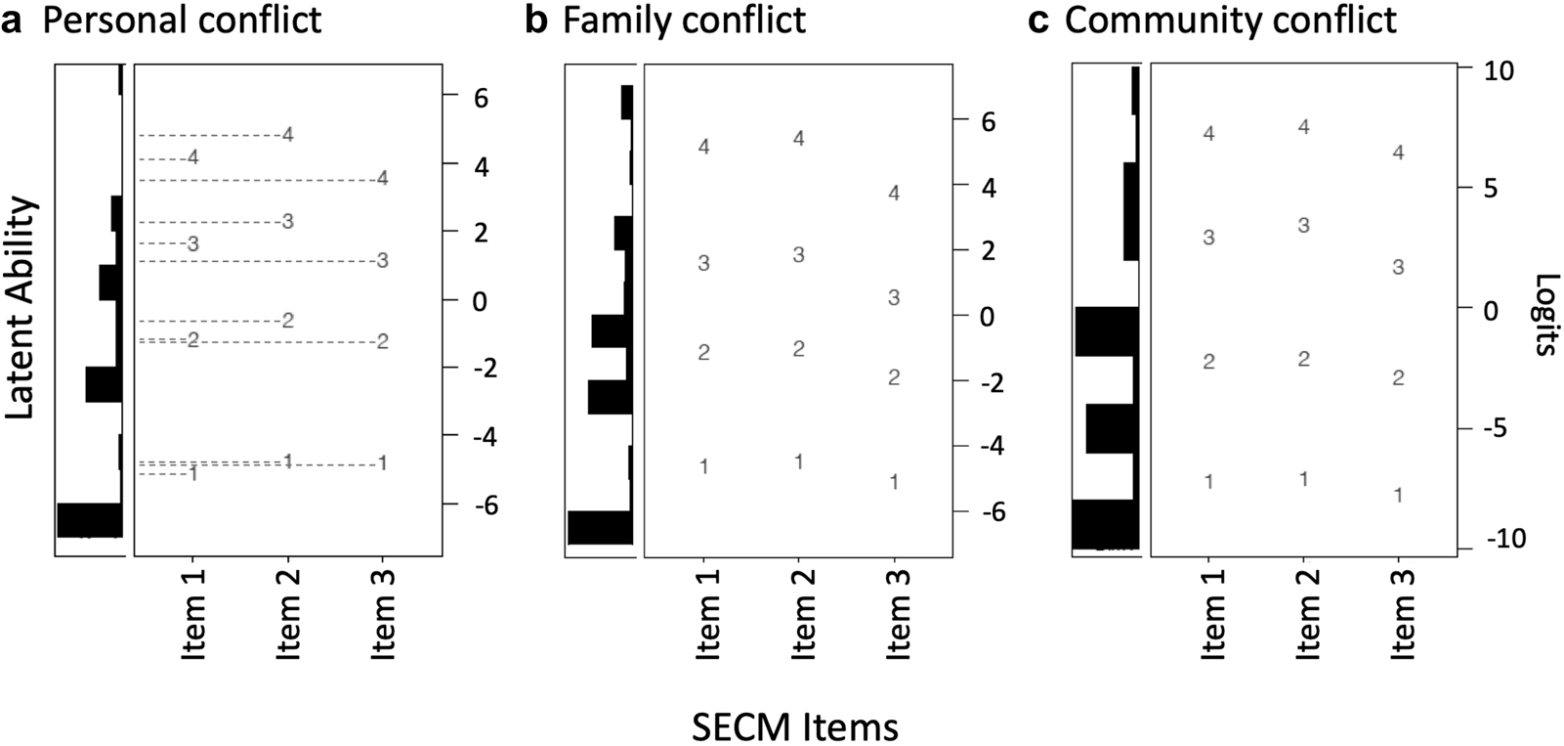
Wright maps for the three scales of the SECM instrument. The numbers in the Wright map represent the locations of the Thurstonian threshold. The dashed lines shown in figure a indicate the locations along the latent trait where measurement can occur (i.e., the “tick marks”)

**Fig. 5:**
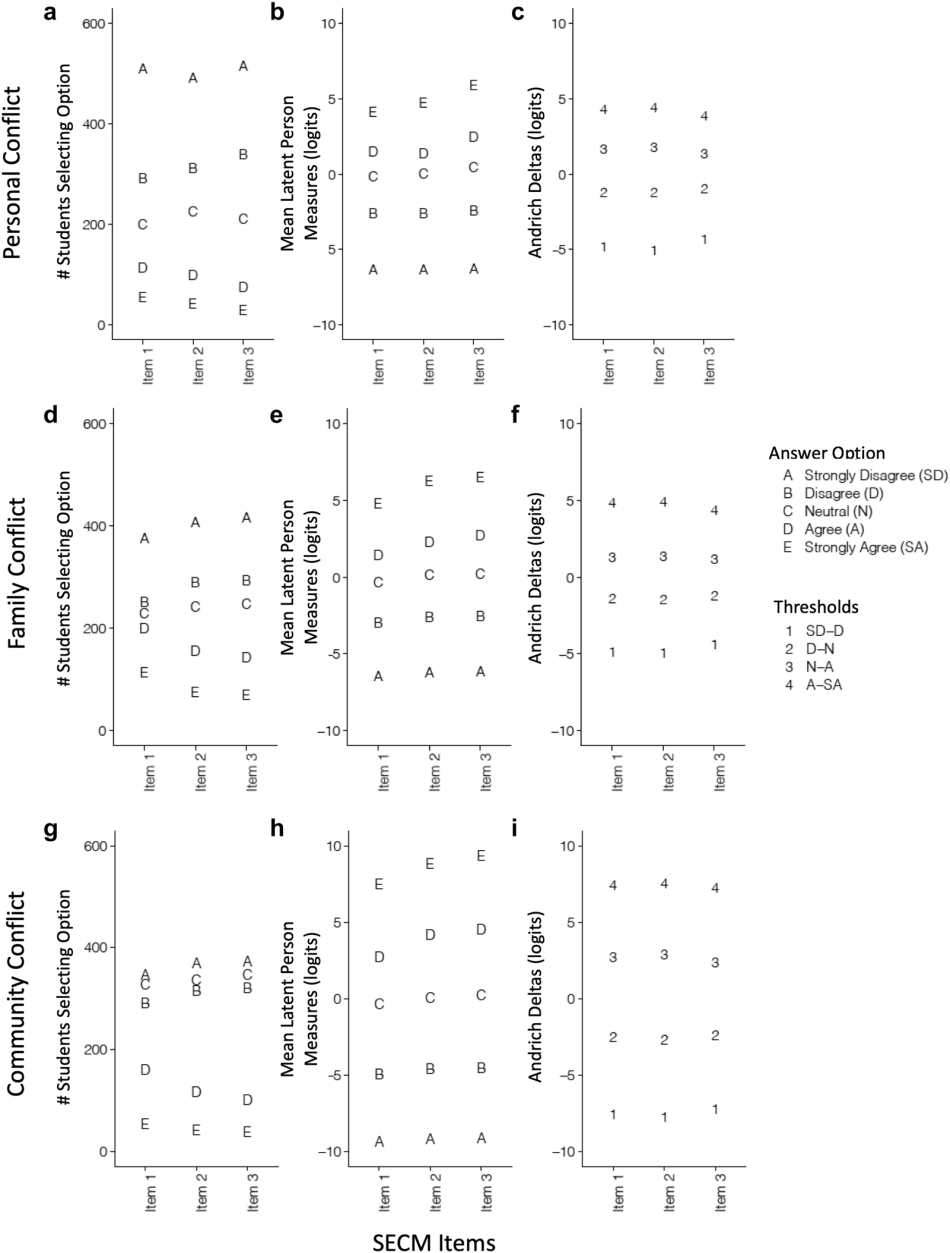
Rating scale functioning for the SECM

The SECM demonstrated acceptable precision and high reliability in its measurement of perceptions of conflict with evolution. The Wright map indicated that respondents were variable in their latent levels of the construct at all scales, and the Thurstonian thresholds spanned much of this variability, generating 10–14 ‘tick marks’ along the latent trait where measurement could occur (Fig. 4). Taken together, the Thurstonian thresholds for all items within each scale were generally well-spaced at the higher end of the trait (i.e., at intermediate and high levels of the perception of conflict with evolution), producing only small gaps in measurement (see thresholds 2–4). However, the thresholds at the lower end of the trait (i.e., at low levels of perceived conflict with evolution) had larger gaps among them, likely resulting in less precise measurement (see thresholds 1–2). Although the precision with which respondents could be clustered into bins at the lower end of the trait might be relatively low as compared to the higher end of the trait, the reliability of the clustering was very high. Specifically, both item and person reliabilities were strong (EAP = 0.867–0.903; WLE = 0.867–0.915), suggesting that items and persons at all levels of the trait could be meaningfully and consistently ordered on a linear scale. Likewise, the rating scale displayed a strong correspondence between participants’ answer choices for each item (Fig. 5b, e, h) and their overall latent person measures, and showed no evidence of disordered thresholds (Fig. 5c, f, i). Collectively, these findings suggest acceptable reliability and precision of measurement for the SECM.

We found no evidence of DIF across time for any scale of evolutionary conflict in the SECM (t-ratio < 2 for all interactions between items and time), suggesting measurement invariance for time in our sample. Therefore, the SECM appeared to measure the same construct pre- and post-course, making pre-post comparisons of the magnitude of the latent trait appropriate.

### RQ2: Are respondents interpreting items as we anticipated?

Approximately 92% (562/619) of respondents indicated that the proposed ‘community’ categories captured the top three most important aspects of their communities. The remaining 8% (57/619) of respondents selected “not applicable” for one of their answer options. It is possible that aspects of their community were missing from the options provided or that two categories sufficiently described the entirety of their communities. Regardless, the vast majority of respondents defined their communities using the categories we defined a priori. Furthermore, although there were some differences among respondents in the specific choices they made, 88% (547/619) of respondents chose friends (from high school or college) as one of the top three most important parts of their community, and this pattern did not differ by their personal conflict level group (*X*^*2*^ = 3.9348, p = 0.047, 95% CI [-0.12, 0.004]). Therefore, in addition to defining their communities as we intended, most respondents also defined their communities similarly to each other, and this definition did not differ significantly based on conflict level.

### RQ3: Are latent SECM measures convergent with measures of similar constructs?

There was a significant positive correlation between the latent measures of each respondent’s perception of their family’s conflict with evolution ideas (i.e., SECM Family item set) and the modified Inclusion of Others in Self [IOS] item (Spearman correlation: r = 0.50, p < 0.001).

### RQ4: Do measures of conflict derived from the SECM contribute to the explanation of evolution acceptance above and beyond the contributions of religiosity and evolution knowledge?

**Fig. 6:**
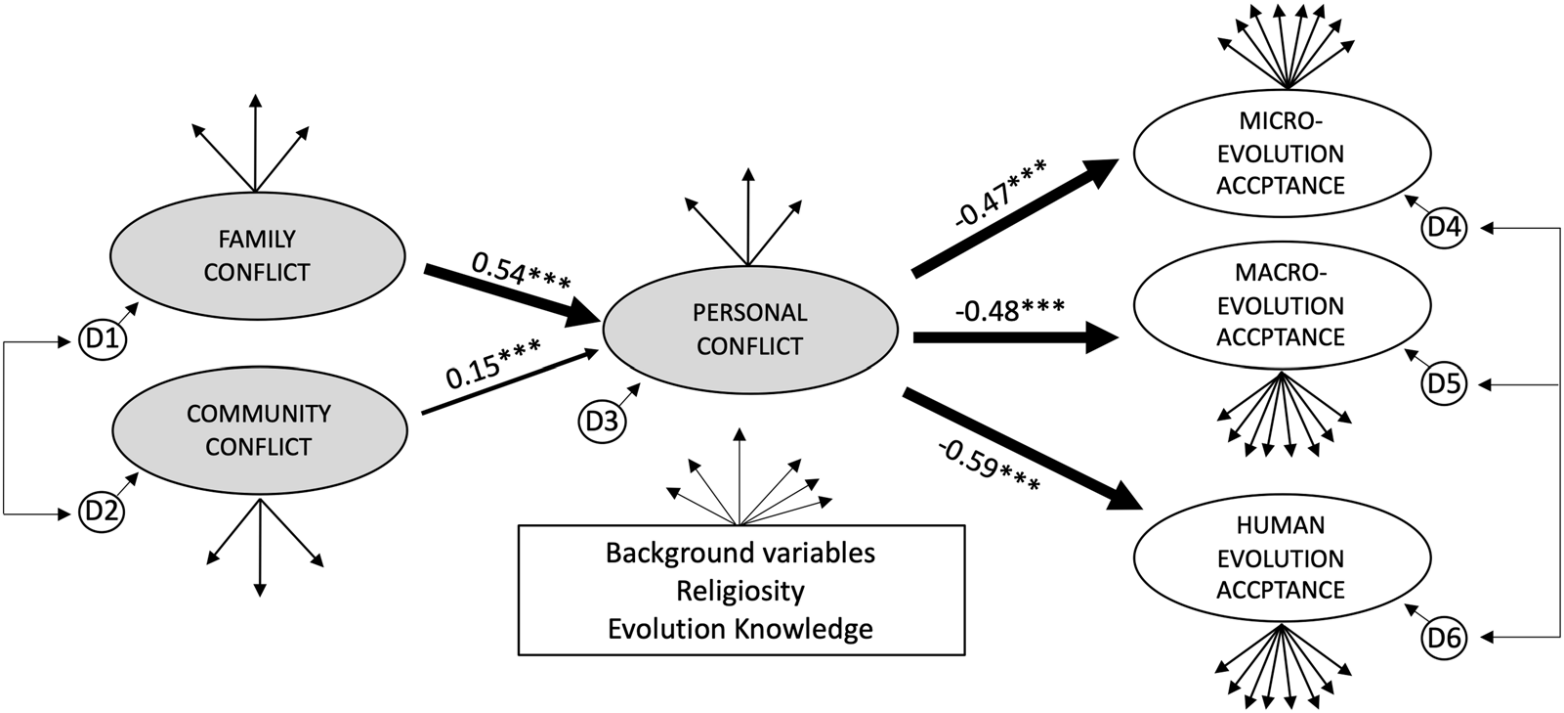
Latent variable path model with standardized path coefficients (B). Evolution knowledge, religiosity, and background variables were modeled as having causal links (to each SECM factor (i.e., personal conflict, family conflict, community conflict) and each I-SEA factor (i.e., microevolution acceptance, macroevolution acceptance, human evolution acceptance) (represented here by a box with arrows emerging from it), which effectively controlled for the effects of these variables on all path coefficients. Assuming a correct underlying model, the path coefficients shown in the model reflect the magnitude of the causal impact of these variables, holding all other variables constant (including evolution knowledge, religiosity, and background variables). The disturbances for each factor are represented by a circled D. Significance levels: * < 0.05; ** < 0.01; *** < 0.001

We used SEM (specifically LVPM) to evaluate the hypothesized causal relationships among the SECM scales, and between the SECM and evolution acceptance (Fig. 6). Our measurement model had acceptable fit to the data (see Table 3) and no posteriori changes to the model were made. Given the acceptable fit of the measurement model to our sample data, we had license to estimate the structural model. The structural model also had acceptable fit to the sample data (see Table 3). See Additional file 1: Table S2 for summary statistics of factor loadings, Additional file 2: Table S3 for the raw variance covariance matrix, and Additional file 3: Table S4 for the means and standard deviations of raw scores.

**Table 3:** Fit statistics from measurement and structural latent variable path models

	Chi-square (df)	SRMR	Robust RMSEA	Robust CFI
Measurement Model	4227.005 (1980)	0.05	0.03	0.99
Structural Model	4815.846 (2467)	0.05	0.03	0.99

Assuming a correct underlying model, the perceptions of family and community positively impacted perceptions of personal conflict with evolution, which subsequently negatively impacted evolution acceptance. Specifically, controlling for all other modeled variables (including background variables, religiosity and evolution knowledge), a one standard deviation increase in perceptions of family conflict and community conflict caused, on average, a 0.54 and 0.15 standard deviation increase in personal conflict, respectively (Fig. 6, Table 4). Subsequently, a one standard deviation increase in personal conflict caused a 0.47–0.59 standard deviation decrease in acceptance (Fig. 6, Table 4). Therefore, personal conflict had a moderately-sized causal impact on all scales of acceptance that was *above and beyond* the contributions of evolution knowledge and religiosity.

**Table 4:** Parameter estimates (unstandardized [β] and standardized [B]) for the latent variable path model

Endogenous variable	Exogenous variable	β	SE	z-value	p-value	B
Personal conflict ~	Family conflict	0.520	0.030	17.440	< 0.001	0.540
Personal conflict ~	Community conflict	0.142	0.030	4.700	< 0.001	0.148
Microevolution acceptance ~	Personal conflict	− 0.311	0.028	− 11.040	< 0.001	− 0.467
Macroevolution acceptance ~	Personal conflict	− 0.321	0.028	− 11.559	< 0.001	− 0.476
Human evolution acceptance ~	Personal conflict	− 0.518	0.031	− 16.731	< 0.001	− 0.588

## Discussion

The purpose of this study was to advance empirical and conceptual work in evolution education by developing an instrument capable of productively measuring perceptions of conflict with evolution (SECM). Below we discuss findings regarding validity evidence and instrument functioning and the potential of the SECM to clarify the factors impacting evolution acceptance. Table 5 summarizes the findings according to validity evidence category.

**Table 5:** Summary of findings linked to validity evidence category

Evidence type	Finding
Evidence based on test content	
Logical analysis	Experts agreed the items were representative of the literature review and the target construct
Evidence based on internal structure	
Item fit	Acceptable
Dimensionality	Three dimensions (personal conflict, family conflict, community conflict) supported
Precision	Acceptable
Reliability	Acceptable
Measurement invariance	Present. Pre-post comparisons of SECM measures would be appropriate
Evidence based on relationships with other variables	
Convergence with measure of similar construct	Family conflict was significantly correlated with a modified IOS item. Evidence of convergence needed for personal and community conflict
Evidence based on response processes	
Respondent cognition related to test	Partially addressed (i.e., “community”). Evidence is also needed to evaluate respondents’ interpretations of items not studied (e.g., using methods such as “think-aloud” interviews)
Validity generalization	
Validity studies in different geographic, institutional, and demographic contexts	Not addressed. Evidence needed to evaluate if the inferences made from the instrument generalize to other contexts
Evidence of consequences	
Outcomes of instrument use	Not addressed. Consequences (e.g., positive, negative) of instrument implementation for respondents and associated educational programs and systems

### Validity evidence and instrument functioning

Multiple scales and variables relating to the perception of conflict with evolution have been proposed in the literature over the past several decades. The development of the SECM involved content domain delineation, construct conceptualization, and operationalization as a measurement tool. An expert panel verified that the structure and items of the SECM appropriately and completely represented the targeted content domain. The potential of the SECM to generate robust measures of the intended construct was examined using six criteria: item fit, reliability, dimensionality, rating scale functioning, person-item alignment, and measurement invariance. The SECM achieved acceptable results for all of these criteria. Specifically, the instrument was found to operate most effectively as three distinct dimensions with items grouped by social scale (i.e., personal, family, community) and not by the variable (i.e., values, culture, beliefs). This finding emerged from the PCA of Rasch residuals, and was further supported by a likelihood ratio test of this three-dimensional structure. Overall, these findings suggest that each scale of conflict operates as a distinct dimension, and the broader social context associated with conflict perception was not adequately captured by one subscale alone.

We also found that the conflict items (i.e., values, culture, and beliefs) within each scale adequately fit the Rasch model and productively measured the construct. The SECM also had high reliability values and a well-functioning rating scale, indicating that the items (by their agreeability) and the persons (by their latent ability) could be consistently and meaningfully ordered on a linear scale, allowing these measures to be analyzed using parametric statistical applications (Boone et al. 2014). The items were also generally well-matched to the target population (i.e., university undergraduates in an introductory biology course) and respondents with intermediate to high measures on the latent trait had a high precision of measurement. Respondents with low measures on the latent trait had relatively lower measurement precision, suggesting that an easier item (i.e., one that students with relatively low latent levels of conflict would endorse) might be a valuable addition to the instrument to increase precision at this portion of the rating scale. However, because precise measurement of the trait where conflict levels are intermediate to high is likely to be more important to researchers than precise measurement where levels are low, we do not consider this finding to be a significant weakness of the SECM.

The items were also found to be measurement invariant across time, suggesting that respondents interpreted items similarly before and after evolution instruction. Therefore, the SECM could be used to make meaningful inferences about changes in perceptions of conflict with evolution in response to instruction or targeted interventions. Several researchers have proposed frameworks for guiding the development of interventions that target the compatibility of evolution and religion (e.g., Barnes and Brownell 2017) and the bounded nature of science (e.g., Nelson et al. 2019). Results from researchers who have implemented curricular interventions aimed at reducing conflict (particularly between religion and evolution) suggest that it is a malleable trait that can be successfully impacted by instruction (e.g., Barnes and Brownell 2017; Nehm and Schonfeld 2007; Truong et al. 2018).

Measures from the family subscale of the SECM correlated significantly and modestly with the Inclusion of Others in Self (IOS) instrument, which measured the perception of compatibility between family and evolutionary ideas. This modest correlation provides convergent validity evidence for the SECM, but also suggests that the perception of conflict (or incompatibility) (targeted by the SECM) and the perception of compatibility (targeted by the IOS) are not necessarily direct opposites of one another from a measurement perspective. More work on the perception of compatibility (or harmony) with evolution could offer different inferences and is clearly warranted.

Finally, the follow-up question asking respondents to share the top three most important parts of their communities indicated that they defined their communities as intended. Furthermore, most respondents defined their communities similarly to each other (regardless of their level of conflict), with friends as one of the most important components of community. Additional substantive validity evidence would be valuable for other aspects of the SECM.

### Using the SECM to advance evolution education research

In order to illustrate how the SECM could advance understanding of the complex web of variables connected to evolution acceptance, SEM was used to evaluate hypothesized causal relationships among the SECM scales, and between the SECM and evolution acceptance. Several other variables shown to be important in the literature (e.g., knowledge, religiosity) were also collected to examine if and how the SECM enhances explanatory insights. Two major findings emerged: (i) family and community conflict both had *unique* causal contributions to personal conflict, with family showing a stronger and modest impact, and (ii) personal conflict had a significant and modest causal impact on all scales of evolution acceptance *above and beyond* the contributions of religiosity, evolution knowledge, and background variables. We discuss the implications of each of these findings below.

It is notable that both family and community conflict had unique casual impacts on personal perceptions of conflict with evolution. Few studies have directly compared the strength of the association of community and family on personal conflict. Studies that have collected data at both scales have similarly found that family contributions were more apparent than community contributions (e.g., Winslow et al. 2011). Such studies have the potential to provide insights into possible targets for intervention development. However, no studies to our knowledge have addressed this relationship in a latent modeling framework, which allows for more precise and accurate measures of each latent trait and the nature of the relationships among them. The SECM is well suited for use in such frameworks. The specific causal structure modeled among the SECM scales in this paper was based on our interpretation of the literature (see above). While the acceptable data-model fit we report suggests that this structure is a tenable explanation for the associations in the data, it is important to emphasize that alternative, mathematically equivalent models that would explain the data equally well may exist (Mueller and Hancock 2008). Therefore, researchers are not limited to modeling the subscales or the items of the SECM in the manner highlighted in this paper, nor are they obligated to use all three subscales simultaneously. The validity evidence presented in this paper was generated for each scale individually so use of select scales is acceptable. Furthermore, the Rasch-based evidence for internal structure validity made no distinction between reflective (i.e., the items are manifestations or indicators of the latent trait) and formative models (i.e., the items cause the latent trait), and researchers may consider alternative relationships between each latent trait and its associated items that best suits their conceptual framework. Overall, alternate theory-driven formulations of the relationships among the scales and the items is encouraged.

As a focus of future study, it is worth considering whether the strength of the causal links among the SECM scales might differ between members of different identity groups. For example, preliminary evidence suggests that the relative impact of family vs. community on personal perspectives may differ across racial groups. Mead et al. (2015) report that the science-related career interests of African American and Latino/a university students were more strongly influenced by people that identify as being members of the same racial/ethnic group, whereas European Americans were more strongly influenced by their parents or guardians. Furthermore, Dewsbury et al. (2019) reports that Latino American university students described both the importance of familial ties, and the sociocultural expectations related to their identities, as key roles in determining their perspectives on STEM-related career choice. Therefore, taken together, these studies suggest that perceptions of community conflict with evolution may be an important contributor to the personal conflict perception of URMs. Furthermore, if the experience of conflict within the classroom is generally agreed to be a problematic feature of the learning environment–and evidence suggests it should be (e.g., Azmitia et al. 2008; Chemers et al. 2011; Goodenow 1993; Goodenow and Grady 1993; Griffith and Brem 2004)–then the differential contributors to personal conflict should be an important target for study and intervention. Reducing perceived conflict with evolution may therefore be an important goal that extends beyond its relationship with evolution acceptance. This is an avenue of research that has not to our knowledge been explored empirically.

Experiencing conflict with a core concept of biology may also be linked to the physical and emotional well-being of students, and as such, might impact psychosocial and performance outcomes. For example, perceptions of conflict may reduce students’ sense of belonging and science identity compatibility in science classrooms. Sense of belonging and identity compatibility can be powerful motivators for academic commitment and achievement (Chemers et al. 2011; Goodenow 1993; Goodenow and Grady 1993). Indeed, feelings of isolation have been shown to be greater for URMs in STEM (Cohen and Garcia 2008) and linked to (i) doubts about their ability to succeed and (ii) stereotype threat activation. Students with religious backgrounds also report feelings of alienation in biology classrooms (Barnes et al. 2017c). These feelings of exclusion can emerge from students’ multiple, and sometimes competing, identities. Experiencing conflict with aspects of one’s identity, as well as developing new identities that might not appear to be compatible with existing ones may be stressful (Azmitia et al. 2008; Griffith and Brem 2004), which may in turn have negative impacts on memory processing and learning (Vogel and Schwabe 2016). For these reasons, conflict may negatively impact students’ well-being and academic performance. We therefore join other authors (e.g., Mead et al. 2015) in advocating for the disaggregation of data in evolution education by key identity variables (such as race) that could contribute to feelings of conflict and exclusion.

We also report that personal conflict had a significant and modest causal impact on all scales of evolution acceptance *above and beyond* the contributions of religiosity, evolution knowledge, and background variables. Human evolution acceptance showed the largest impact of personal conflict, which indicated that while the perception of conflict with evolution was important for all scales of evolution acceptance, it may be most important for the acceptance of human evolution. These results suggest that the perception of conflict with evolution may in fact impact evolution acceptance in a manner that is not accounted for by other available variables. These results do not suggest that all theoretically important variables have been accounted for by this model. For example, religious denominational differences could be an important contributor to perceptions of conflict.^2^ As a next step, researchers could consider incorporating the SECM into more complicated SEM models that treat evolution knowledge and religiosity (as well as other theoretically important variables, such as religious denomination [see Jensen et al. 2019,3]) as part of the structural pathways leading to and emerging from acceptance. Such a model would allow for the direct comparison of the strength of the causal relationships of each variable with acceptance, and provide valuable insights into the most worthwhile targets for the development of interventions. Therefore, the SECM allows for the testing of hypotheses about the causes of conflict, which extend far beyond what we have presented in this study.

### Limitations

The SECM is a first (and admittedly incomplete) step towards more completely operationalizing and measuring conflict with evolution; different conceptual frameworks and measurement approaches than we have used may be equally valuable. Although our psychometric work was fairly comprehensive, validity comprises many categories that were not investigated in our study (AERA et al. 2014). Indeed, the process of instrument validation involves an iterative (and often gradual) accumulation of multiple forms of evidence that collectively support the interpretations of instrument measures for their proposed uses. Our study used the framework of construct validity (Campbell and Nehm 2013; Messick 1995). In line with this framework, we generated four forms of validity evidence: content validity, internal structure validity, convergent validity, and substantive validity. While these forms of validity evidence have so far supported the theoretical underpinnings of the SECM and the quality of the inferences that it generates, the instrument would benefit from additional evidence for these and other uninvestigated forms. In particular, convergent evidence was presented for only one of the three subscales of the SECM (i.e., family conflict). The other two subscales are in need of this form of evidence as well. Moreover, evidence based on response processes (i.e., substantive validity) have only been studied for the community scale, and validity generalization (i.e., generalization validity) and evidence of consequences have not been studied. Evidence based on response processes addresses the cognitive processes involved in answering questions (AERA et al. 2014). “Think-aloud” interviews may be used for this purpose in order to evaluate if respondent interpretations match intended item meanings for other items and scales. This form of validity evidence can help answer questions about the different interpretations respondents from different identity groups could have. Evidence based on validity generalization deals with the extent to which validity evidence generated in one setting can be generalized to new settings (AERA et al. 2014). At present, the SECM has been administered and evaluated at one type of institution. Therefore, validity studies on the SECM in different geographic, institutional, and demographic settings are needed. Without evidence of generalizability, this instrument should not yet be interpreted in other settings without researchers producing local validity evidence (AERA et al. 2014). Evidence based on consequences deals with the extent to which the outcomes or benefits proposed by the test are realized (AERA et al. 2014). For example, if decreasing the perception of conflict with evolution decreases anxiety, increases feelings of belonging in the classroom, and increases evolution acceptance, then reducing conflict through targeted interventions should display these outcomes. Furthermore, if low evolution acceptance and the perception of conflict with evolution are barriers for students persisting in biology majors and seeking evolution-related careers, level of conflict should be a significant indirect or direct predictor of these outcomes. Clearly, this study is one very small step towards advancing work on measuring conflict with evolution.

## Conclusion

This paper introduced the SECM, an easy-to-administer instrument designed to measure perceptions of conflict with evolution at multiple scales. The SECM embodies three attributes discussed in the literature: (i) it addresses the hierarchical nature of human social structures (conceptualized as personal, family, and community), (ii) probes conflict as it relates to human values, cultures, and beliefs, (iii) allows conflict to encompass diverse sources (not only religion). Four forms of validity evidence–content, internal structure, convergent, and substantive–supported the meaning of the inferences drawn from SECM measures. The instrument was found to be representative of the intended construct, capable of producing reliable and precise measures, and aligned with measures of a similar construct. The SECM was designed to be used flexibly so that researchers may apply it in a manner that best aligns with their hypotheses and analytical frameworks. Specifically, the subscales may be implemented separately or together, and used in several theory-driven orientations. Generating robust measures of the perception of conflict with evolution using the SECM has the potential for advancing understanding of the factors accounting for evolution acceptance, and for informing practices and outcomes relating to evolution education.

## Supplementary information

**Additional file 1: Table S1.** Fit statistics, reliabilities, and dimensionality of the I-SEA, CANS, and Religiosity instruments. **Table S2.** Factor loadings and variance extracted for the SECM, I-SEA, CANS, and Religiosity instruments.

**Additional file 2: Table S3.** Variance-covariance matrix.

**Additional file 3: Table S4.** Means and standard deviations of raw scores.

## Data Availability

Summary data are available in the supplement (i.e., variance–covariance matrix, means and standard deviations of raw item-level data). All other data will be available from the authors upon request.

## Footnotes

1 Walker et al. introduced multiple new measures in place of existing, more robustly validated measures without explanation. Psychometric and methodological limitations of the study suggest caution is needed when interpreting the findings.

2 We thank an anonymous reviewer for this suggestion.

3 The findings of Jensen et al. 2019 should be interpreted with caution because they (i) introduced multiple new measures in place of existing, more robustly validated measures without explanation. (ii) performed exploratory and causal modeling on the same data set, (iii) interpreted the SEM model despite unacceptable model fit. Psychometric and methodological limitations of the study suggest caution is needed when interpreting the findings.

## References

[CR1] AERA, APA, NCME (2014). Standards for educational and psychological testing.

[CR2] Adams RJ, Wu ML, Wilson M (2012). The Rasch rating model and the disordered threshold controversy. Educ Psychol Meas.

[CR3] Andrich D (2013). An expanded derivation of the threshold structure of the polytomous Rasch model that dispels any ‘‘threshold disorder controversy’’. Educ Psychol Meas.

[CR4] Aron A, Aron EN, Smollan D (1992). Inclusion of other in the self scale and the structure of interpersonal closeness. J Pers Soc Psychol.

[CR5] Azmitia M, Syed M, Radamacher K. On the intersection of personal and social identities: introduction and evidence from a longitudinal study of emerging adults. In: Azmitia M, Syed M, Radmacher K, editors. The intersections of personal and social identities. New directions for child and adolescent development. San Francisco: Jossey-Bass; 2008, vol. 120, pp 1–16. 10.1002/cd.212 18521867

[CR6] Bailey G, Han J, Wright D, Graves JL (2011). Religiously expressed fatalism and the perceived need for science and scientific process to empower agency. Int J Sci Soc.

[CR7] Barnes ME, Dunlop HM, Sinatra GM, Hendrix TM (2020). “Accepting evolution means you can’t believe in god”: atheistic perceptions of evolution among college biology students. CBE Life Sci Educ.

[CR8] Barnes ME, Brownell SE (2017). A call to use cultural competence when teaching evolution to religious college students: introducing religious cultural competence in evolution education (ReCCEE). CBE Life Sci Educ.

[CR9] Barnes ME, Elser J, Brownell SE (2017). Impact of a short evolution module on students’ perceived conflict between religion and evolution. Am Biol Teacher.

[CR10] Barnes ME, Evans EM, Hazel A, Brownell SE, Nesse RM (2017). Teleological reasoning, not acceptance of evolution, impacts students’ ability to learn natural selection. Evol Educ Outreach..

[CR11] Barnes ME, Truong JM, Brownell SE (2017). Experiences of Judeo-Christian students in undergraduate biology. CBE Life Sci Educ.

[CR12] Barone LM, Petto AJ, Campbell BC (2014). Predictors of evolution acceptance in a museum population. Evol Educ Outreach..

[CR13] Bond TG, Fox CM (2001). Applying the Rasch model: Fundamental measurement in the human sciences.

[CR14] Boone WJ (2016). Rasch analysis for instrument development: why, when, and how?. CBE Life Sci Educ.

[CR15] Boone WJ, Staver JR, Yale MS (2014). Rasch analysis in the human sciences.

[CR16] Borsboom D, Mellenbergh GJ, van Heerden J (2005). The theoretical status of latent variables. Psychol Rev.

[CR17] Brem SK, Ranney M, Schindel J (2003). Perceived consequences of evolution: college students perceive negative personal and social impact in evolutionary theory. Sci Educ.

[CR18] Causadias JM (2018). Do we overemphasize the role of culture in the behavior of racial/ethnic minorities? Evidence of a cultural (mis)attribution bias in American psychology. Am Psychol.

[CR19] Coyne JA (2015). Faith versus fact: why science and religion are incompatible.

[CR20] Campbell CE, Nehm RH (2013). A critical analysis of assessment quality in genomics and bioinformatics education research. CBE Life Sci Educ.

[CR21] Chemers MM, Zugriggen EL, Syed M, Goza B, Bearman S (2011). The role of efficacy and identity in science career commitment among underrepresented minority students. J Soc Issues.

[CR22] Clark SL, Dyar C, Maung N, London B (2016). Psychosocial pathways to STEM engagement among graduate students in the life sciences. CBE Life Sci Educ.

[CR23] Clough MP (1994). Diminish students' resistance to biological evolution. Am Biol Teacher.

[CR24] Cohen GL, Garcia J (2008). Identity, belonging, and achievement: a model, interventions, implications. Curr Direct Psychol Sci.

[CR25] Cohen AB, Shariff AF, Hill PC (2008). The accessibility of religious beliefs. J Res Pers.

[CR26] de Ayala RJ, Hancock GR, Mueller RO (2019). Item response theory. The reviewer’s guide to quantitative methods in the social sciences.

[CR27] Dagher ZR, BouJaoude S (1997). Scientific views and religious beliefs of college students: the case of biological evolution. J Res Sci Teach.

[CR28] Dawkins R (2009). The god delusion.

[CR29] Deniz H, Donnelly LA, Yilmaz I (2008). Exploring the factors related to acceptance of evolutionary theory among Turkish preservice biology teachers: toward a more informative conceptual ecology for biological evolution. J Res Sci Teach.

[CR30] Dewsbury BM, Taylor C, Reid A, Viamonte C (2019). Career choice among first-generation, minority STEM college students. J Microbiol Biol Educ.

[CR31] Donnelly LA, Kazempour M, Amirshokoohi A (2009). High school students’ perceptions of evolution instruction: acceptance and evolution learning experiences. Res Sci Educ.

[CR32] Dunk RDP, Petto AJ, Wiles JR, Campbell BC (2017). A multifactorial analysis of acceptance of evolution. Evol Educ Outreach.

[CR33] Gelfand MJ, Nishii LH, Raver JL (2006). On the nature and importance of cultural tightness–looseness. J Appl Psychol.

[CR34] Glaze AL, Goldston MJ, Dantzler J (2014). Evolution in the southeastern USA: factors influencing acceptance and rejection in pre-service science teachers. Int J Sci Math Educ.

[CR35] Goodenow C, Grady KE (1993). The relationship of school belonging and friends' values to academic motivation among urban adolescent students. J Exp Educ.

[CR36] Goodenow C (1993). Classroom belonging among early adolescent students: relationships to motivation and achievement. J Early Adolesc.

[CR37] Graves J (2019). African Americans in evolutionary science: where we have been, and what's next. Evol Educ Outreach.

[CR38] Griffith JA, Brem SK (2004). Teaching evolutionary biology: pressures, stress, and coping. J Res Sci Teach.

[CR39] Grigg K, Manderson L (2016). The Australian racism, acceptance, and cultural-ethnocentrism scale (RACES): item response theory findings. Int J Equity Health.

[CR40] Ha M, Baldwin BC, Nehm RH (2015). The long-term impacts of short-term professional development: science teachers and evolution. Evol Educ Outreach.

[CR41] Ha M, Haury DL, Nehm RH (2012). Feeling of certainty: uncovering a missing link between knowledge and acceptance of evolution. J Res Sci Teach.

[CR42] Ha M, Wei X, Wang J, Hou D, Nehm RH (2019). Chinese pre-service biology teachers’ evolutionary knowledge, reasoning patterns, and acceptance levels. Int J Sci Educ.

[CR43] Hambleton RK, Jones RW (1993). An NCME instructional module on comparison of classical test theory and item response theory and their applications to test development. Educ Measure Issues Pract.

[CR44] Heddy BC, Nadelson LS (2012). A global perspective of the variables associated with acceptance of evolution. Evol Educ Outreach.

[CR45] Hill JP (2014). Rejecting evolution: the role of religion, education, and social networks. J Sci Study Relig.

[CR46] Hitlin S (2003). Values as the core of personal identity: drawing links between two theories of the self. Soc Psychol Q.

[CR47] Hitlin S, Piliavin JA (2004). VALUES: reviving a dormant concept. Annu Rev Sociol.

[CR48] Jensen JL, Manwaring KF, Gill RA (2019). Religious affiliation and religiosity and their impact on scientific beliefs in the United States. Bioscience.

[CR49] Kahan DM, Braman D, Gastil J, Slovic P, Mertz CK (2007). Culture and identity-protective cognition: explaining the white-male effect in risk perception. J Empir Leg Stud.

[CR50] Kalinowski ST, Leonard MJ, Taper ML (2016). Development and validation of the Conceptual Assessment of Natural Selection (CANS). CBE Life Sci Educ.

[CR51] Konnemann C, Höger C, Asshoff R, Hammann M, Rieß W (2018). A role for epistemic insight in attitude and belief change? Lessons from a cross-curricular course on evolution and creation. Res Sci Educ.

[CR52] Leung K, Bond MH, de Carrasquel SR, Muñoz C, Hernández M, Murakami F, Yamaguchi S, Bierbrauer G, Singelis TM (2002). Social axioms: the search for universal dimensions of general beliefs about how the world functions. J Cross Cult Psychol.

[CR106] Linacre JM. Category disordering (disordered categories) vs. threshold disordering (disordered thresholds). In: Rasch Measurement Transactions. Institute for Objective Measurement; 1999. https://www.rasch.org/rmtbooks.htm. Accessed 6 Nov 2018.

[CR53] Linacre M, Wright B. Constructing linear measures from counts of qualitative observations. Paper presented at the Fourth International Conference on Bibliometrics, Informetrics and Scientometrics, Berlin; 1993.

[CR54] Liu X (2010). Using and developing measurement instruments in science education: a Rasch modeling approach Charlotte.

[CR55] Lombrozo T, Thanukos A, Weisberg M (2008). The importance of understanding the nature of science for accepting evolution. Evol Educ Outreach..

[CR56] Lommen MJJ, van de Schoot R, Engelhard IM (2014). The experience of traumatic events disrupts the measurement invariance of a posttraumatic stress scale. Front Psychol.

[CR101] MacQueen KM, McLellan E, Metzger DS, Kegeles S, Strauss RP, Scotti R, Blanchard L, Trotter RT (2001). What is community? An evidence-based definition for participatory public health. American Journal of Public Health..

[CR57] Maio GR, Olson JM, Bernard MM, Luke MA, DeLamater J (2003). Ideologies, values, attitudes, and behavior. Handbook of social psychology.

[CR58] Manwaring KF, Jensen JL, Gill RA, Bybee SM (2015). Influencing highly religious undergraduate perceptions of evolution: Mormons as a case study. Evo Edu Outreach.

[CR59] Marini MM, Borgatta EF, Montgomery RJV (2000). Social values and norms. Encyclopedia of sociology.

[CR102] Mead LS, Clarke JB, Forcino F, Graves JL (2015). Factors influencing minority student decisions to consider a career in evolutionary biology. Evol EducOutreach..

[CR61] Messick S (1995). Validity of psychological assessment: validation of inferences from persons’ responses and performances as scientific inquiry into score meaning. Am Psychol.

[CR62] Metzger K, Montplaisir D, Haines D, Nickodem K (2018). Investigating undergraduate health sciences students’ acceptance of evolution using MATE and GAENE. Evol Educ Outreach.

[CR63] Michener HA, DeLamater J, Myers D (2004). Social Psychology.

[CR64] Mikulić J, Ryan C (2018). Reflective versus formative confusion in SEM based tourism research: a critical comment. Tour Manag.

[CR65] Mueller RO, Hancock GR, Hancock GR, Mueller RO (2019). Structural equation modeling. The reviewer’s guide to quantitative methods in the social sciences.

[CR66] Mueller RO, Hancock GR, Osborne J (2008). Best practices in structural equation modeling. Best practices in quantitative methods.

[CR67] Nadelson LS, Southerland S (2012). A more fine-grained measure of student’s acceptance of evolution: Development of the Inventory of Student Evolution Acceptance–I-SEA. Int J Sci Educ.

[CR103] Nehm RH, Ha M (2011). Item feature effects in evolution assessment. Journal of Research in Science Teaching..

[CR69] Nehm RH, Mead LS (2019). Evolution assessment: introduction to the special issue. Evol Educ Outreach.

[CR70] Nehm RH, Schonfeld IS (2007). Does increasing biology teacher knowledge of evolution and the nature of science lead to greater preference for the teaching of evolution in schools?. J Sci Teacher Educ.

[CR68] Nehm RH, Kim SY, Sheppard K (2009). Academic preparation in biology and advocacy for teaching evolution: biology versus non-biology teachers. Sci Educ.

[CR104] Nehm RH, Beggrow EP, Opfer JE, Ha M (2012). Reasoning about natural selection: diagnosing contextual competency using the ACORNS Instrument. TheAmerican Biology Teacher..

[CR71] Nelson CE, Scharmann LC, Beard J, Flammer LI (2019). The nature of science as a foundation for fostering a better understanding of evolution. Evol Educ Outreach.

[CR72] Neumann I, Neumann K, Nehm R (2010). Evaluating instrument quality in science education: Rasch-based analyses of a Nature of Science Test. Int J Sci Educ.

[CR73] Opfer JE, Nehm RH, Ha M (2012). Cognitive foundations for science assessment design: knowing what students know about evolution. J Res Sci Teach.

[CR74] Paz-y-Miño CG, Espinosa A (2011). New England faculty and college students differ in their views about evolution, creationism, intelligent design, and religiosity. Evol Educ Outreach..

[CR75] Paz-y-Minos G, Espinosa A (2009). Acceptance of evolution increases with student academic level: a comparison between a secular and religious college. Evol Educ Outreach..

[CR76] Rissler LJ, Duncan SI, Caruso NM (2014). The relative importance of religion and education on university students’ views of evolution in the Deep South and state science standards across the United States. Evol Educ Outreach..

[CR77] Robitzsch A, Kiefer T, Wu M. TAM: Test analysis modules. R package version 2.10. https://CRAN.R-project.org/package=TAM; 2018.

[CR105] Romine WL, Walter EM, Bosse E, Todd AN (2017). Understanding patterns of evolution acceptance—a new implementation of the measure of acceptance of the theory of evolution. Journal of Research in Science Teaching..

[CR78] Rosseel Y. lavaan: Latent Variable Analysis. R package version 0.6–6. https://cran.r-project.org/web/packages/lavaan/index.html; 2020a.

[CR79] Rosseel Y (2020). The lavaan tutorial.

[CR80] Sbeglia GC, Nehm RH (2018). Measuring evolution acceptance using the GAENE: Influences of gender, race, degree plan, and instruction. Evol Educ Outreach..

[CR81] Sbeglia GC, Nehm RH (2019). Do you see what I-SEA? A Rasch analysis of the psychometric properties of the inventory of student evolution acceptance. Sci Educ.

[CR82] Scharmann LC, Harris WM (1992). Teaching evolution: understanding and applying the nature of science. J Res Sci Teach.

[CR83] Schwartz SH, Bilsky W (1987). Toward a psychological structure of human values. J Pers Soc Psychol.

[CR84] Schwartz SH, Zanna MP (1992). Universals in the content and structure of values: theoretical advances and empirical tests in 20 countries. Advances in experimental social psychology.

[CR85] Shin JEL, Levy SR, London B (2016). Effects of role model exposure on STEM and non-STEM student engagement. J Appl Soc Psychol.

[CR86] Sinclair A, Pendarvis MP, Baldwin B (1997). The relationship between college zoology students’ beliefs about evolutionary theory and religion. J Res Dev Educ.

[CR87] Smith MU, Siegel H, McInerney JD (1995). Foundational issues in evolution education. Sci Educ.

[CR88] Southerland SA, Sinatra GM, Matthews MR (2001). Belief, knowledge, and science education. Educ Psychol Rev.

[CR89] Taras V, Rowney J, Steel P (2008). Half a century of measuring culture: review of approaches, challenges, and limitations based on the analysis of 121 instruments for quantifying culture. J Int Manage.

[CR90] Tropp LR, Wright SC (2001). In-group identification as the inclusion of in-group in the self. Pers Soc Psychol Bull.

[CR91] Truong JM, Barnes ME, Brownell SE (2018). Can six minutes of culturally competent evolution education reduce students’ level of perceived conflict between evolution and religion?. Am Biol Teacher.

[CR92] Turner FM (1978). The Victorian conflict between science and religion: a professional dimension. Isis.

[CR93] Van Riper CJ, Kyle GT (2014). Understanding the internal processes of behavioral engagement in a national park: a latent variable path analysis of the value-belief-norm theory. J Environ Psychol.

[CR94] van de Schoot R, Schmidt P, De Beuckelaer A, editors. Measurement Invariance. Lausanne: Frontiers Media. 2015; doi: 10.3389/978-2-88919-650-0. 10.3389/fpsyg.2015.01064 PMC4516821 26283995

[CR95] Vogel S, Schwabe L (2016). Learning and memory under stress: implications for the classroom. NPJ Sci Learn.

[CR96] Walker JD, Wassenberg D, Franta G, Cotner S (2017). What determines student acceptance of politically controversial scientific conclusions?. Res Teach.

[CR97] Winslow MW, Staver JR, Scharmann LC (2011). Evolution and personal religious belief: Christian university biology-related majors’ search for reconciliation. J Res Sci Teach.

[CR98] Wright BD (1996). Comparing Rasch measurement and factor analysis. Struct Equ Model Multidiscipl Jo.

[CR99] Xiao YJ, Coppin G, Van Bavel JJ (2016). Perceiving the world through group-colored glasses: a perceptual model of intergroup relations. Psychol Inq.

[CR100] Yang Y, He P, Liu X (2017). Validation of an instrument for measuring students’ understanding of interdisciplinary science in grades 4–8 over multiple semesters: A Rasch measurement study. Int J Sci Math Educ.

