# Supplementary material for: Illuminating the complexities of conflict with evolution: validation of the scales of evolutionary conflict measure (SECM)
Source: Evolution (N Y). 2020 Nov 23;13(1):23. doi: 10.1186/s12052-020-00137-5 (PMC7683450; doi:10.1186/s12052-020-00137-5)
Supplement: Supplementary file 1 — Additional file 1: Table S1. Fit statistics, reliabilities, and dimensionality of the I-SEA, CANS, and Religiosity instruments. Table S2. Factor loadings and variance extracted for the SECM, I-SEA, CANS, and Religiosity instruments. [file 12052_2020_137_MOESM1_ESM.docx]

**Supplemental Table 1:** Fit statistics, reliabilities, and dimensionality of the I-SEA, CANS, and Religiosity instruments.

|  | # items | Fit Statistics Range  (Outfit/Infit) | Reliability  (Item/person) | PCA of Rasch residuals (eigenvalue of 1^st^ contrast) |
| --- | --- | --- | --- | --- |
| **I-SEA** |  |  |  |  |
| Micro | 8 | 0.86-1.27/0.97-1.23 | 0.84/0.75 | 2.19 |
| Macro | 8 | 0.85-1.18/0.87-1.16 | 0.82/0.74 | 1.67 |
| Human | 8 | 0.79-1.23/0.86-1.33 | 0.89/0.91 | 2.7 |
| **CANS** | 24 | 0.73-1.40/0.78-1.23 | 0.80/0.76 | 2.37 |
| **Religiosity** | 9 | 0.66-1.81^a^/0.70-1.50 | 0.93/0.92 | 2.13 |

^a^One item on the religiosity instrument (item 9) was outside of the range that is informative for measurement, but it was also not degrading to measurement, and was thus retained.

**Supplemental Table 2:** Factor loadings and variance extracted for the SECM, I-SEA, CANS, and Religiosity instruments.

|  | **Mean Standardized Factor Loadings** | **Variance Extracted^a^** |
| --- | --- | --- |
| SECM |  |  |
| Personal | 0.95 | 0.90 |
| Family | 0.95 | 0.90 |
| Community | 0.96 | 0.92 |
| I-SEA |  |  |
| Micro | 0.71 | 0.51 |
| Macro | 0.67 | 0.45 |
| Human | 0.76 | 0.58 |
| CANS | 0.50 | 0.31 |
| Religiosity | 0.89 | 0.83 |

^a^Variance extracted is the sum of the squared standardized factor loadings divided by the number of indicators (Mueller and Hancock 2019). It represents the average variance of the factor explained by the items.

**Supplemental Table 3:** Variance-covariance matrix.

**Supplemental Table 4:** Means and standard deviations of raw scores.
